# Evolutionary comparison of prenylation pathway in kinetoplastid *Leishmania* and its sister *Leptomonas*

**DOI:** 10.1186/s12862-015-0538-3

**Published:** 2015-11-21

**Authors:** Indira Singh Chauhan, Jaspreet Kaur, Shagun Krishna, Arpita Ghosh, Prashant Singh, Mohammad Imran Siddiqi, Neeloo Singh

**Affiliations:** Biochemistry Division, CSIR Central Drug Research Institute, Jankipuram Extension, Sitapur Road, Lucknow, 226031 India; Department of Biochemistry, Shri Ram Murti Smarak Institute of Medical Sciences, Bareilly, 243202 India; Molecular and Structural Biology Division, CSIR Central Drug Research Institute, Jankipuram Extension, Sitapur Road, Lucknow, 226031 India; Xcelris Genomics, Ahmedabad, India; Department of Chemistry, Dayanand Anglo Vedic (P.G.) College, Dehradun, 248001 India

**Keywords:** Leishmania donovani, Leptomonas, Prenylation pathway

## Abstract

**Background:**

*Leptomonas* is monogenetic kinetoplastid parasite of insects and is primitive in comparison to *Leishmania*. Comparative studies of these two kinetoplastid may share light on the evolutionary transition to dixenous parasitism in *Leishmania*. In order to adapt and survive within two hosts, *Leishmania* species must have acquired virulence factors in addition to mechanisms that mediate susceptibility/resistance to infection in the pathology associated with disease. Rab proteins are key mediators of vesicle transport and contribute greatly to the evolution of complexity of membrane transport system. In this study we used our whole genome sequence data of these two divergent kinetoplastids to analyze the orthologues/paralogues of Rab proteins.

**Results:**

During change of lifestyle from monogenetic (*Leptomonas*) to digenetic (*Leishmania*), we found that the prenyl machinery remained unchanged. Geranylgeranyl transferase-I (GGTase-I) was absent in both *Leishmania* and its sister *Leptomonas.* Farnesyltransferase (FTase) and geranylgeranyl transferase-II (GGTase-II) were identified for protein prenylation. We predict that activity of the missing alpha-subunit (α-subunit) of GGTase-II in *Leptomonas* was probably contributed by the α-subunit of FTase, while beta-subunit (β-subunit) of GGTase-II was conserved and indicated functional conservation in the evolution of these two kinetoplastids. Therefore the β-subunit emerges as an excellent target for compounds inhibiting parasite activity in clinical cases of co-infections. We also confirmed that during the evolution to digenetic life style in *Leishmania*, the parasite acquired capabilities to evade drug action and maintain parasite virulence in the host with the incorporation of short-chain dehydrogenase/reductase (SDR/MDR) superfamily in Rab genes.

**Conclusion:**

Our study based on whole genome sequences is the first to build comparative evolutionary analysis and identification of prenylation proteins in *Leishmania* and its sister *Leptomonas.* The information presented in our present work has importance for drug design targeted to kill *L. donovani* in humans but not affect the human form of the prenylation enzymes.

**Electronic supplementary material:**

The online version of this article (doi:10.1186/s12862-015-0538-3) contains supplementary material, which is available to authorized users.

## Background

Visceral leishmaniasis (VL) is endemic in 98 countries with 350 million people at risk around the world and 300,000 are infected/year (http://www.dndi.org/diseases/leishmaniasis.php). More than 90 % of visceral leishmaniasis (VL) occurs in seven countries: India, Bangladesh, Nepal, Sudan, Ethiopia, Kenya and Brazil (http://www.dndi.org/diseases/leishmaniasis.php). Leishmaniasis is caused by protozoan parasites of *Leishmania* genus. VL also known as kala-azar in India, is the most severe form of leishmaniasis (http://www.dndi.org/diseases-projects/diseases/vl.html) and anthroponotic transmission (human to vector to human) occurs. Whole genome sequencing of clinical isolates of *Leishmania donovani* and *Leptomonas* has previously been completed in our lab [1]. An important insight gained through this effort was the confirmation of co-infection of *Leptomonas* with the visceralizing *Leishmania* species. This disturbing trend has been reported so far only in India [1–3] hinting towards zoonotic spread of the disease and indicates that *Leptomonas* may be a new pathogen. In such a situation the question remains that a changed paradigm in chemotherapy should be adopted targeting the co-infections.

**Fig. 1:**
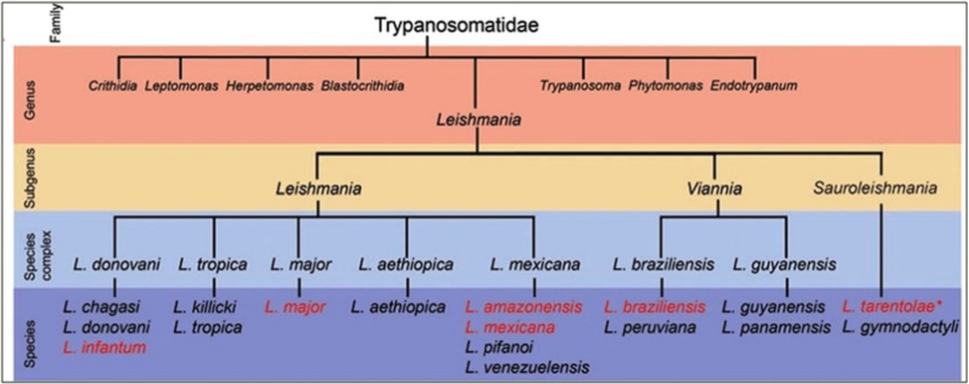
Overview of the *Leishmania* complex; classification of the *Leishmania* genus, subgenus and species complex (F. Real et al. 2013) [61]. Both kinetoplastids *Leishmania donovani* and *Leptomonas* arose from a common ancestor

*Leptomonas* is monogenetic kinetoplastid parasite of insects and primitive in comparison to *Leishmania* [1] and these two parasites arose from a common ancestor as shown in Fig. 1 [4, 5]. At some time during the evolution, *Leishmania* appears to have lost the ability to be transmitted in nature from invertebrate host to another and adapted to a life cycle alternating between invertebrate and vertebrate host [6, 7]*.* With the availability of complete genome sequence with us of these two kinetoplastids [1] representing important evolutionary branch points viz *Leishmania* and *Leptomonas*, in this study we have analyzed the enzymes prenyltransferases (farnesyltransferase and geranylgeranyltransferase) of these kinetoplastids. Cellular organization and signaling in both unicellular and multicellular organisms is heavily influenced by the Ras superfamily of small GTP-binding proteins. These proteins have a structurally and mechanistically preserved GTP-binding core despite considerable divergence in sequence and function [8]. Protein trafficking pathways are frequently exploited by human pathogens to gain entry and survive within host cells [9]. Studying the evolution of protein trafficking is essential to understand the origins of eukaryotes. Among trafficking-associated proteins, the Rab family expanded most in evolution, suggesting that it provided the primary diversification element in the evolution of trafficking [10]. The prenylation pathway remains largely unexplored in Kinetoplastida. Therefore, it was of interest for us to carry out the phylogenetic profiling of Rab geranylgeranyltransferase (RabGGTase) and Rab proteins in both *Leishmania* and its sister *Leptomonas*. In this study we used a classification and phylogeny based approach to define subfamilies of RabGGTase and Rab proteins, we built evolutionary trees using maximum likelihood and distance based methods. We provide a comprehensive view of RabGGTase and Rab evolution for sister *Leptomonas* and with various *Leishmania* species. Since, drugs that inhibit enzymes involved in protein prenylation could be interesting antiparasitic agents; we used one specific known inhibitor of RabGGTase to predict the structure activity relationship with the parasite enzyme.

## Results and discussion

### Kinetoplastid genome phylogeny

**Fig. 2:**
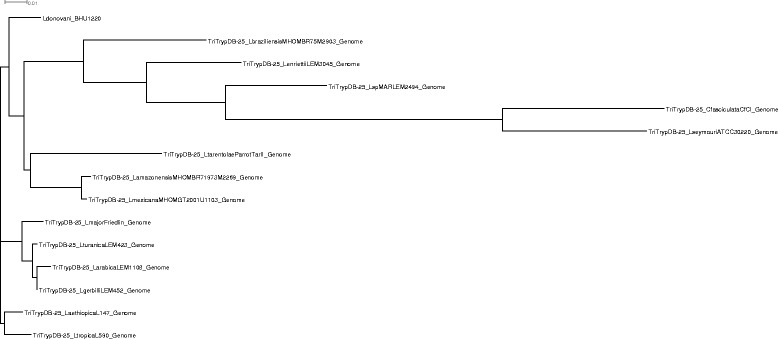
Kinetoplastid genome phylogeny. A cladogram depicting kinetoplastid phylogenetic relationships among genome of *Crithidia fasciculate, Leishmania aethiopica, Leishmania amazonensis, Leishmania arabica , Leishmania braziliensis, Leishmania enriettii, Leishmania gerbilli, Leishmania donovani, Leishmania major, Leishmania Mexicana, L. sp. MAR LEM2494, Leishmania tarentolae, Leishmania tropica , Leishmania turanica and Leptomonas seymouri*

We compared the genomes of *Leishmania* species to *Leptomonas* in order to obtained information on kinetoplastid phylogeny (Fig. 2). *Crithidia* was taken as an outgroup. We observed that the *L. donovani* isolate from India was placed at the crown in comparison to *Leptomonas.*

### *Leishmania donovani* and *Leptomonas* farnesyltransferase

**Fig. 3:**
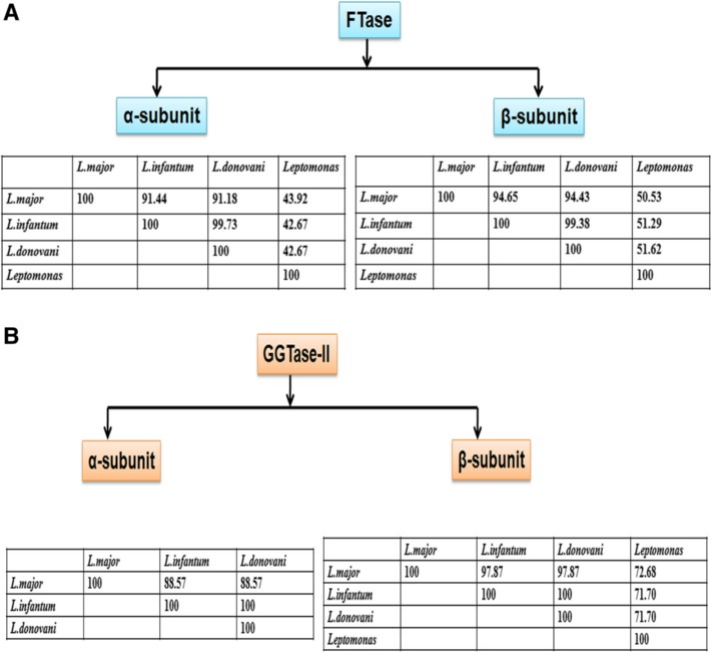
**a** Percent identity of the α and β-subunit of FTase enzyme in *Leishmania* species and its sister *Leptomonas.* **b** Percent identity of the α and β-subunit of GGTase-II enzyme in *Leishmania* species and its sister *Leptomonas*

In *Leishmania donovani*, α-subunit of farnesyltransferase (LdFTase alpha) was located on chromosome 29 (LdBPK_292070) with accession number XP_003862625.1. In *Leptomonas* α-subunit of FTase (*Leptomonas* FTase alpha) was identified from the contig (contig_2652) of *Leptomonas* genome. Using ClustalW, percent identity of the α-subunit of LdFTase and *Leptomonas* FTase with *L. major* (XP_003722277.1) and *L. infantum* (XP_001466722.1) was summarized in Fig. 3a and it can be seen that the region of sequence identity was found extensively at the C terminus (Additional file 1: Figure S1).

β-subunit of FTase in *Leishmania donovani* (LdFTase beta) was located on chromosome 26 (LdBPK_261450) with accession number XP_003861732.1. In *Leptomonas,* β-subunit of FTase (*Leptomonas* FTase beta) was identified from the contig (contig_1135) of *Leptomonas* genome. Using ClustalW, percent identity of the β-subunit of LdFTase and *Leptomonas* FTase with *L. major* (XP_001684151.1) and *L. infantum* (XP_001470492.1) was summarized in Fig. 3a. In comparison with the α-subunit of FTase among *L. major, L. infantum* and *L. donovani* the β-subunit showed more sequence identity (Additional file 1: Figure S2). It is also reported that the β-subunit of prenyltransferases are more conserved than α-subunit [11]. The β-subunit appears to have invariable domain architecture throughout evolution and its invariable nature is due to functional rather than structural constraints.

### *Leishmania donovani* and *Leptomonas* geranylgeranyltransferase

Using ClustalW, percent identity of the α-subunit of LdGGTase-II (XM_001468149) with *L. major* (XM_001685808) and *L. infantum* (XP_001468186) was summarized in Fig. 3b. α-subunit of this enzyme was totally conserved between the two visceral species *L. donovani* and *L. infantum* but quite variable between the cutaneous *L. major* and visceral species (Additional file 1: Figure S3). Non conservative amino acids alanine and serine at positions 82, 88, 214, 440 and 235, of the visceral species were replaced by valine and threonine in the cutaneous species respectively (Additional file 1: Figure S3). It has been reported by Peterson et al. that parasites harboring a pair of point mutations from Ala-16 to Val-16 and from Ser-108 to Thr-108 are resistant to cycloguanil [12]. A similar substitution in α-subunit of GGTase-II could be responsible for its survival and multiplication within the host cell. No sequence identity was observed to α-subunit of GGTase-II in *Leptomonas* genome with *L. major* (XM_001685808), *L. infantum* (XP_001468186) and LdGGTase-II (XM_001468149).

In our study, the β-subunit of GGTase-II of *L. donovani* was totally conserved in both the *Leishmania* species responsible for the visceral manifestation of the disease viz. *L. infantum* (XP_001468743) and *L. donovani* (XP_003864545). The carboxy terminal of this enzyme was completely conserved between the visceral (*L. infantum*, XP_001468743) and cutaneous species (*L. major*, XP_001686510) of *Leishmania* (Additional file 1: Figure S4) however in the N-terminal, differences in the cutaneous species were observed. Non conservative amino acids aspartate and glycine at positions 57 and 61 of the visceral species were substituted in the cutaneous species (Additional file 1: Figure S4). The susceptibility to infection by *Leishmania NRAMP1* gene has been similarly reported [13] to be substitution in non conservative amino acid aspartate and glycine. A similar substitution in β-subunit of GGTase-II maybe due to the different requirement for mode of protein trafficking.

**Fig. 4:**
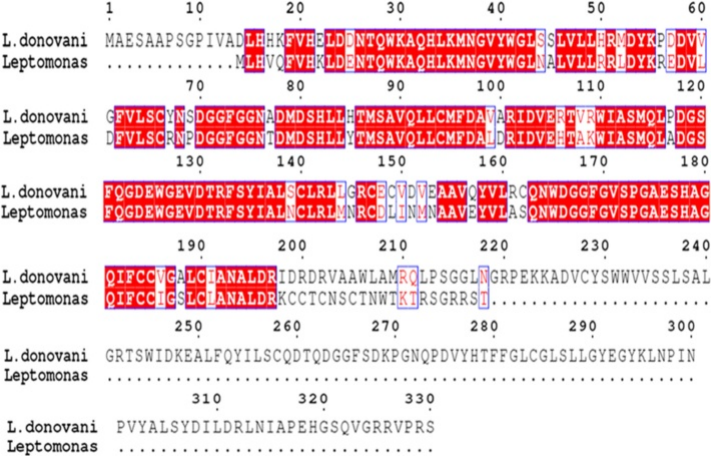
Multiple sequence alignment of GGTase-II (β-subunit) of *Leishmania donovani* (accession number XP_003864545) with its sister *Leptomonas* (contig_5507) showing conserved regions (red colored) based on percentage identity

Percent identity of the β-subunit of GGTase-II in *Leptomonas* to Old World *L. major* (XP_001686510) and *L. infantum* (XP_001468743) is shown (Figs. 3b and Fig. 4). New World, *L. panamensis* (XP_010698581) and *L. braziliensis* (XP_001564646), showed 81 % identity with *Leptomonas* whereas in *L. donovani* identity with only Old World was observed.

**Fig. 5:**
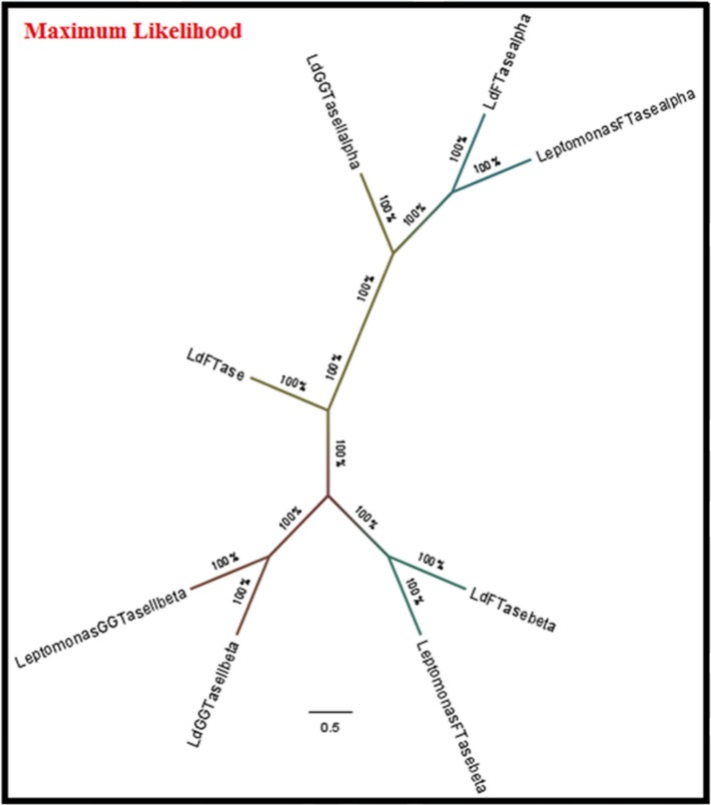
We reconstructed the evolutionary relationships between FTase and GGTase-II of *Leishmania* and *Leptomonas* by using Neighbor-Joining method

The evolutionary relationship between FTase and GGTase-II of *Leishmania* and *Leptomonas* was reconstructed by using Neighbor-Joining method (Fig. 5). In our study, α-subunit of FTase between *Leishmania* and *Leptomonas* were in close phylogeny. But as regards to the holoenzyme, α-subunit was placed very differently to the β-subunit of these enzymes. We concluded that α-subunit of GGTase-II is almost similar to the corresponding α-subunit of FTase in both kinetoplastids. β-subunit of GGTase-II is very similar to the corresponding β-subunit of FTase in both kinetoplastids.

### Rab sequence mining in *L. major* and *L. infantum*

Among the prenylation proteins, Rabs are small GTP-binding proteins which are the largest family within the Ras superfamily. These act as membrane associated molecular switches and regulate vesicular trafficking [14–16]. The number of Rab proteins and gene complexity in an organism has been correlated with multicellularity of organism. A multicellular organism will require/have higher number of Rabs encoded by its genome [17]. *Plasmodium falciparum* has been shown to encode 11 Rabs, *Toxoplasma gondii* encodes 15 Rabs, *Leishmania* encodes 12 Rabs, *Drosophila melanogaster* encodes 29 Rabs, and *Homo sapiens* encodes > 60 Rab proteins, which correlates with their multicelluarity and organism structural complexity.

Until now, despite the importance of the intracellular vesicle transport in *Leishmania* pathogenicity, very few publications are available regarding number, diversity, complexity and functions of *Leishmania* Rab gene family [18–20]. Therefore, it was of interest for us to carry out the phylogenetic profiling of the Rab repertoire in the lower eukaryote *Leishmania.* The exhaustive mining of *Leishmania* Rab proteins in GeneDB (www.genedb.org) and NCBI (http://www.ncbi.nlm.nih.gov/) database revealed many full length proteins, some were annotated as Rabs of various families, but most were annotated as putative Rabs or Rab like proteins, in this aspect the *L. major* Rabs are better annotated compared to *L. infantum* Rabs. Our search retrieved 14 completely annotated Rabs in *L. major* whereas 18 Rabs are reported in *L. major* by Berriman et al. [21]. Similarly in *L. infantum*, we found 13 proteins annotated as Rabs and few other proteins annotated as putative Rab or Rab like proteins. The details of Rabs of both *L. infantum* and *L. major*, along with name, accession number, gene ID and chromosomal location in Additional file 2: Table S1.

### Rab sequence mining in *Leishmania* and *Leptomonas*

**Fig. 6:**
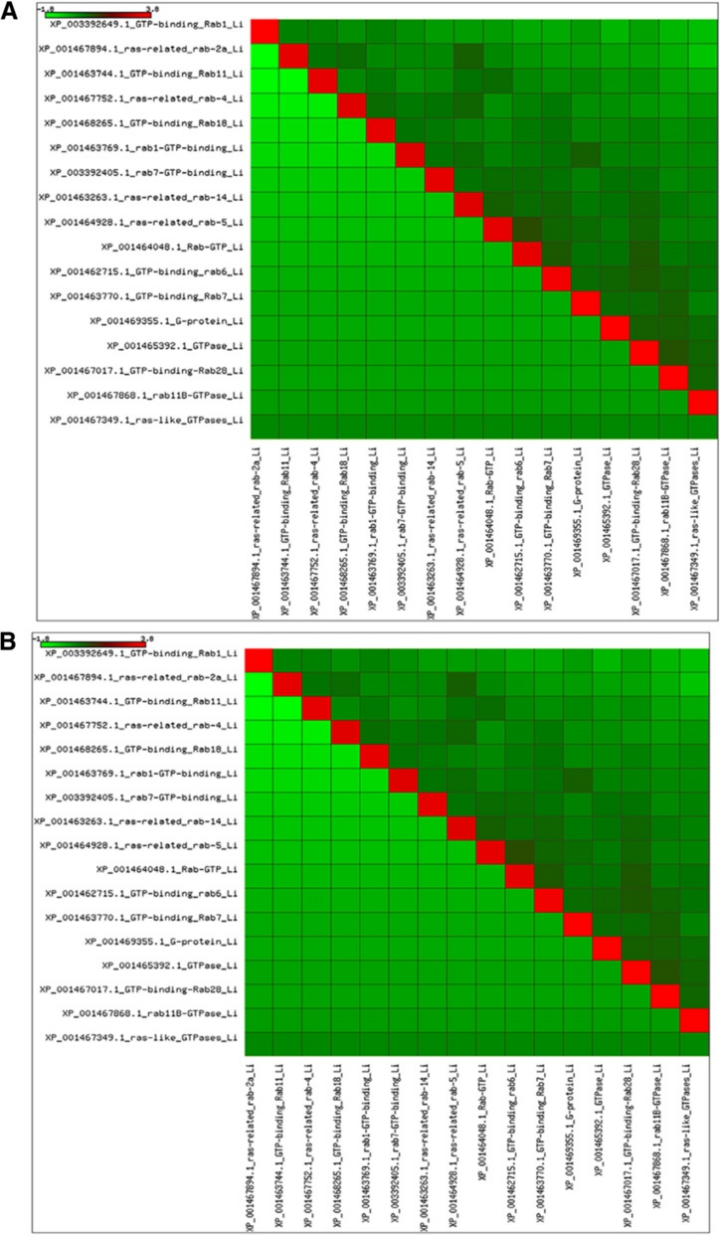
Heat-map depicting percent identity between **a** *L. major* and **b** *L. infantum*

From our whole genome sequence of *L. donovani* and *Leptomonas* some Rabs were annotated as Rabs of various families, but most were annotated as putative Rabs or Rab like proteins. We retrieved 13 completely annotated Rabs in *L. donovani* (Additional file 1: Figure S5)*.* Similarly in *Leptomonas*, we found 11 proteins annotated as Rabs (Additional file 1: Figure S6). The details of Rabs of both *L. donovani* and *Leptomonas* are presented in Additional file 2: Table S2 and S3 respectively. A pair wise comparison of the Rab proteins retrieved from the *L. major* (Fig. 6a) and *L. infantum* (Fig. 6b), *L. donovani* (Additional file 2: Table S4A) and *Leptomonas* (Additional file 2: Table S4B) were calculated and phylogenetic tree was constructed using the Rabs orthologues sequences of *L. major, L. infantum, L. donovani* and *Leptomonas* (Additional file 1: Figure S7).

To visualize the synteny in Rab genes between *Leishmania donovani* and *Leptomonas*, we constructed a Circos plot linking the homologous Rab genes (Additional file 1: Figure S8) The Rab genes of *Leptomonas* are represented on the left side and Rab genes of *Leishmania donovani* are represented on the right side. Rab4, Rab7 GTP and Rab18 showed extensive synteny in both the species. Rab1 and Rab7 of *Leishmania donovani* have no similarity with any genes in *Leptomona*s. Whereas few genes in *Leishmania donovani* are partially similar such as Rab28, Rab14, Rab11B, Rab11, Rab6, Rab5, Rab2A and GTP binding Rab1 to *Leptomonas*.

Thirteen annotated Rabs of *L. major* and *L. infantum* were exactly the same as in *L. donovani*, only Rab 21 was present in *L. major* but was not identified in either of the visceral *Leishmania*. What does Rab 21 do? The explanation for this has been addressed in the Phylogeny section. In *Leptomonas* 11 Rabs could be annotated by us, all of them same as found in *Leishmania*.

### Phylogenetic analysis of Rab GTPase

An important role in evolution is played by small GTPases belonging to the Ras superfamily. Rab proteins form the largest branch of the Ras superfamily. This family is much diversified [8]. Each Rab protein has a distinct subcellular location and is responsible for a specific transport step [22, 23]. Rab proteins are key regulators of intracellular vesicular transport and membrane trafficking in exocytic and endocytic pathways [14–16]. The evolution of Rab proteins has been analyzed extensively in Trypanosomes [24] but not in *Leishmania*. We believe that molecular phylogenetic analysis of Rab proteins would provide a unique opportunity for finding the relationship between evolution of *Leishmania* and *Leptomonas. Leishmania* is believed to have evolved from *Leptomonas* where life cycle changed from monogenetic to digenetic [1]. Our study is the first extensive data mining and phylogenetic construction of clinical isolates of *Leishmania* and *Leptomonas* which is based on annotation of Rabs obtained from our whole genome sequencing [1].

**Fig. 7:**
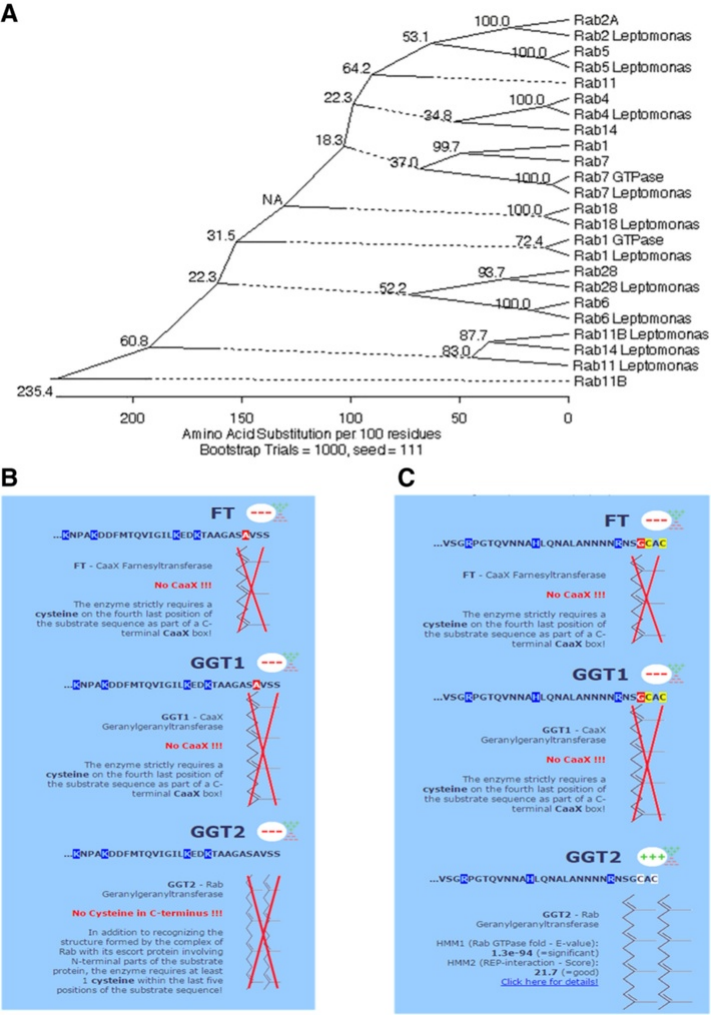
**a** Phylogenetic relationship between Rab sequences of *Leishmania* and *Leptomonas* which is based on multiple sequence alignment (ClustalW algorithm in MEGALIGN with 1000 bootstraps). A dotted line on a phenogram indicates a negative branch length, a common result of averaging and Seed 111 indicates “Random Seed: This option makes bootstrapping consistent with the Clustal interface. The random seed is the value that initializes the pseudo-random number generator. Choosing the same seed causes the generator to produce exactly the same sequence of numbers for a given set of distances and hence the same result. Choosing a different seed value produces an alternative sequence of numbers which should introduce some variation in the result, but it should be similar. The highly conserved values show which parts of the tree should be given greater confidence”. PrePS analysis (PrePS- Prenylation Prediction Suite, http://mendel.imp.ac.at/sat/PrePS/index.html) between Rab 2A and Rab11B of *Leishmania* showed that **b** Rab11B lacks the cysteine residue at C terminus of CAAX signal sequences which is essential for prenylation. **c** Rab 2A has cysteine residue at C terminus of CAAX signal sequences

The orthologues which correspond to genes separated by divergence of the *Leishmania* Rab superfamily in comparison with *Leptomonas* is shown in Fig. 7a. We found that the core Rab repertoire associated with basic endocytosis and exocytosis is fully conserved [25], the endomembrane system appears to be very ancient and maintained same for *Leishmania* and *Leptomonas*, whilst gene duplication has facilitated the building of additional, lineage-specific complexity [24].

Out of 20 Rabs reported to be present in the last eukaryotic common ancestor (LECA) [26], five Rabs are said to be present in any well characterized genome. The core set includes a conserved mechanism for ER to Golgi transport (Rab1 and Rab2), trafficking through the early endosome (Rab5), recycling (Rab4 and Rab11), delivery to the late endosome/lysosome (Rab7) and retrograde transport through the Golgi complex (Rab6) [24]. Inspection of the tree in Fig. 7a reveals that major groups of Rab proteins in the *Leishmania* genome can be broadly related to *Leptomonas* Rabs by homology thus indicating similar function and/or subcellular localization. This co-segregation of Rab GTPases according to common functions rather than to taxonomic relationships indicates a conserved mechanism of Rab interaction with regulators/effectors across evolution [23]. Orthologues of Rabs in *Leishmania* and *Leptomonas* e.g. Rab2, Rab4, Rab5, Rab6, Rab7, Rab18 and Rab28 co-segregated with a strong bootstrap support. This observation suggests that strict phylogeny of function in the Rab superfamily has been maintained and steps required for the basic vesicular transport were almost same in both these kinetoplastids. These core set of Rabs also appear to be orthologues of mammalian Rabs [17]. Remarkably high level sequence conservation is retained between these two kinetoplastids and higher eukaryote orthologues. Parasites (*T. bruci, T. curci* and *L. major*) have ~ 20 Rabs gene and most of which are clearly orthologues with metazoan Rabs [21]. Rab28 is ancient Rab protein that arose before the speciation event separating trypanosomes from the mammalian lineage [24] and is maintained in the kinetoplastid *Leishmania* and its sister species *Leptomonas*. Rab8 is lost in kinetoplastids [26].

Non-essentiality of the different Rab isoforms was observed in both the kinetoplastid genomes but which is observed to be a major contribution in the Rab repertoire of other organisms. We found isoform of Rab2 (2A) and its orthologues with *Leptomonas* in the crown position of phylogenetic tree. Isoform of Rab11 (11B) in *Leishmania* and *Leptomonas* takes basal position in the tree. PrePS analysis (PrePS- Prenylation Prediction Suite, http://mendel.imp.ac.at/sat/PrePS/index.html) of these protein showed that Rab11B lacks the cysteine residue at C terminus of CAAX signal sequences which is essential for prenylation (Fig. 7b). On blastp analysis with the well annotated human Rab family, this Rab11B of *L. donovani*, showed 58 % identity with Rab like protein 2A isoform X7 (XP_006712281.1). Therefore we determined that the Rab11B as annotated in *Leishmania* and *Leptomonas* genome is actually not Rab11B but an ancient isoform of Rab2A. Maybe this protein has conserved GTPase activity and as it evolved to Rab2A (crown position) it acquired specific prenylation activity (Fig. 7c). Alternatively spliced transcript variants encoding different isoforms have been reported. Evolutionary distance of the lineage requires experimental evidence to further support these sequence data. It has been established by Diniz et al. [27]: that Rab2 and Rab11B (which we now identify to be isoform of Rab2A) are actin interacting proteins (AIP) in *L. infantum* and *L. major*.

Rab5 is an early endosome marker as established by Real et al. [28, 29]: in *Leishmania major* and *Leishmania amazonensis*. From our study we found only Rab5 annotation in the genomes of both the kinetoplatids. In *L. donovani* Rab5 has been shown to be is involved in hemoglobin endocytosis [20]. Two isoforms of Rab5 (Rab5A and Rab5B) were reported by them. The strain used by Singh et al. [20]: was UR6 which is a very long term laboratory cultivated avirulent strain and has been established to be *L. tropica* [30] whereas our study of Rab annotations is based on whole genome sequences of recently cultivated clinical isolates of *L. donovani* and *Leptomonas.* On blastp analysis of the Rab5 of both *L. donovani* and *Leptomonas* with human (taxid 9606, accession number NP_002859.1) showed 45 % and 50 % identity to isoform 5B respectively and Rab5b may act as a marker for early endosomes in *L. donovani* [31]. No isoform A in the genome sequence of these two kinetoplastids was present thereby indicating only Rab5 orthologues.

Rab1 GTPase and Rab7 GTPase are actually true Rab1 and Rab7 respectively on the basis of bastp analysis of human Rab family and these were identified as orthologues in both *L. donovani* and *Leptomonas.* Rab7 and Rab9 are two related subfamilies, which show overlapping localization to late endosomes [23]. Rab 9 was absent in the kinetoplastid genomes. Rab9 protein has not yet been reported in unicellular eukaryotes, plants and fungi [23]. Rab7 subfamily belongs to the ancestral set of Rab and arose before the radiation of eukaryotes. Rab7b isoforms are found only in representatives of amphibians, birds and mammals [23]. The Rab1 and Rab7 identified only in *Leishmania* were branched together which suggests paralogous gene duplications. This is a major force in evolution which provides new gene function. Retroposition or lack of introns, as observed in kinetoplastids, is a major contributor for these phenomena. However on blastp analysis with human Rab family, these showed identity 43 and 37 % to Rab 13 and Rab 43 respectively.

RAB11 was found to be a useful marker to track flagellar pocket division and to follow mitochondrial DNA (kinetoplast) segregation. *Leishmania major* antibody raised against *T. brucei* RAB11 which also cross-reacts with the *L. major* homologous [32]. Rab14, appears to regulate endo- lysosomal fusion in *Dictyostelium* [33] primarily found on the contractile vacuole network of membranes, an organelle important in osmotic regulation and, in lesser amounts, in the endo-lysosomal pathway [34].

Rabs in intracellular parasites like *Leishmania* are associated with pathogenesis, as vesicle transport is essential for extracellular nutrient acquisition, release of virulence factors, microbicidal resistance and evasion of host immune responses [19], these requirements being different in the monogenetic ancestor *Leptomonas* hence the difference in phylogeny between *Leishmania* and *Leptomonas* for some Rabs like Rab 11 and Rab 14.

In our study Rab 4 and 14 in *Leishmania* are paralogues and possibly associated with endosomal recycling. Rabs sharing similar functions and/or locations are more closely related than Rab proteins with distinct functions [35]. Rab3 and Rab27, two examples of tissue-restricted Rabs [22] with specialized functions are rightly not detected in our genomes. Rabs are involved in resistance to chemotherapeutic drugs [36]. Rab4 interacts with P-gp, (P-glycoprotein), a large transmembrane protein localized at the plasma membrane that extrudes anticancer drugs and thus decreases their concentration, and toxicity inside the cells [37]. Rab8 has also been reported to be involved in resistance [38].

Flow cytometry experiments showed that sensitivity to doxorubicin was associated with increased drug accumulation in cells expressing the RAB6A variant. WTH3 gene’s product is homologous to the Rab6 and Rab6c genes [38]. WTH3 is a house-keeping gene and its product is capable of binding to GTP molecules. WTH3 [37] and Rab5 [39] both are important genes involved in the cellular multi drug resistance (MDR) phenotype development. It is a well established fact that the H locus of *Leishmania* codes for a short chain dehydrogenase (SDR) gene that is involved in antilfolate resistance [40]. We found SDR to be present in Rab4, Rab6 and Rab18 of *Leishmania* but not in the corresponding Rab genes of *Leptomonas.* A study in our lab has provided evidence supporting the important role played in drug resistance by the Rab6 gene of *Leishmania* having SDR at C terminus of gene (manuscript in communication).

### Structure activity relationship of *L. donovani* GGTase-II with specific inhibitor

Since most prenylated proteins in cells are modified by geranylgeranyl moiety rather than farnesyl [41] it offers Rab prenylation as a lucrative target for chemotherapeutic intervention in *Leishmania* cell. Impairment of Rabs (a GGTase-II substrate) geranylgeranylation by isoprenoid pathway inhibitors (lovastatin, zoledronate and DGBP) have been demonstrated [42]. NE10790 is a phosphonocarboxylate analogue of the potent bisphosphonate risedronate. NE10790 inhibited incorporation of [(14)C] mevalonic acid into Rab but not into H-Ras or Rap1, proteins that are modified by FTase and GGTase I, respectively [43]. NE10790 therefore appears to be the first specific inhibitor of Rab prenylation.

**Fig. 8:**
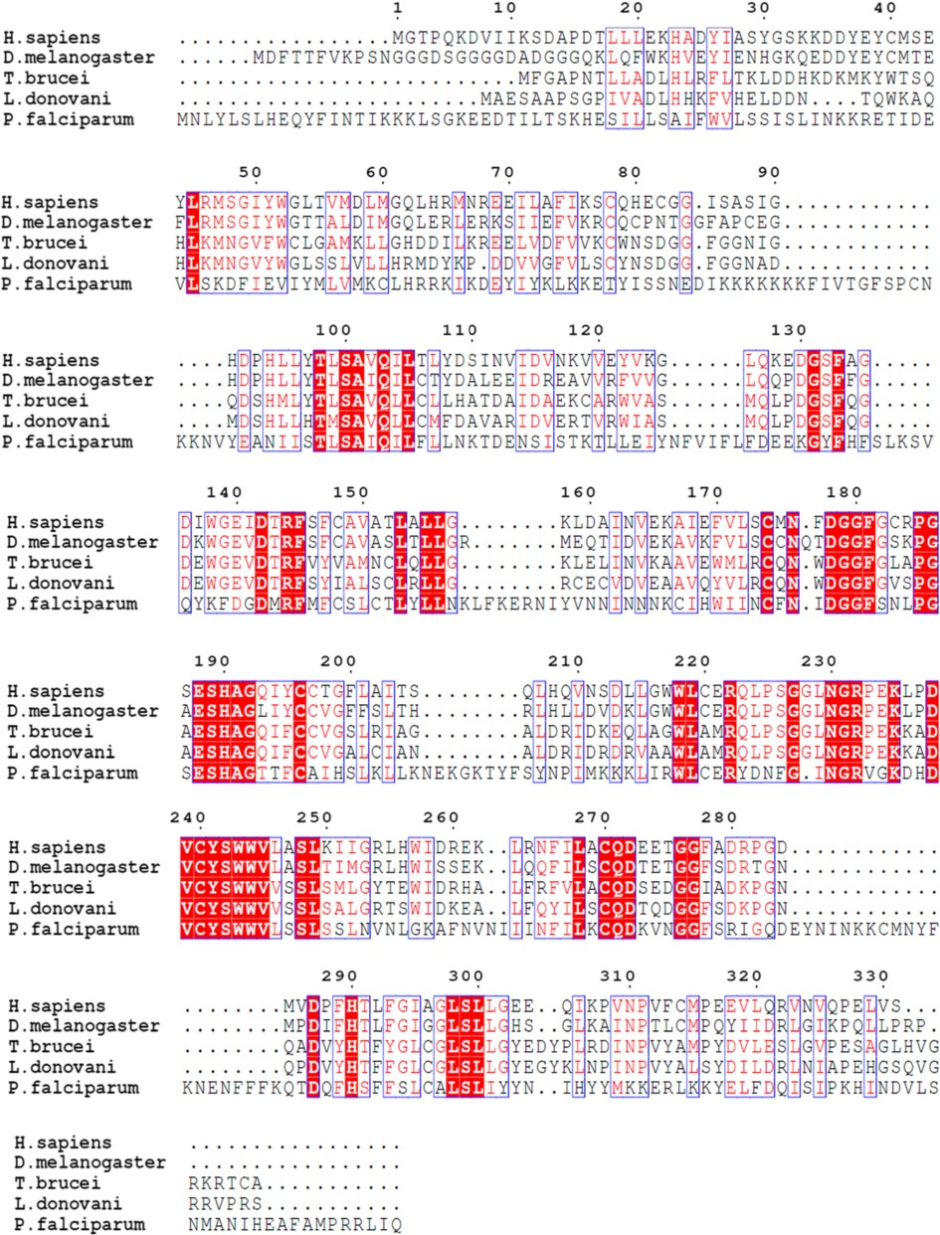
The active site architecture of GGTase-II (β-subunit) of *L. donovani* was compared with other organisms by alignment. Multiple sequence alignment of GGTase-II (β-subunit) of *Leishmania donovani* (accession number XP_003864545) with GGTase-II (β-subunit) of *Homo sapiens* (NP_004573.2), *Plasmodium falciparum* (ACT90629.1), *Trypanosoma brucei* (AAX80948.1) and *Drosophila melanogaster* (AAF51183.1) showing conserved regions (red colored) based on percentage identity

The active site architecture of GGTase-II (β-subunit) of *L. donovani* was compared with other organisms by alignment. The protein sequences of GGTase-II (β-subunit) of *Leishmania donovani* were aligned with the GGTase-II (β-subunit) sequences of *Homo sapiens* (NP_004573.2), *Plasmodium falciparum* (ACT90629.1), *Trypanosoma brucei* (AAX80948.1) and *Drosophila melanogaster* (AAF51183.1). Sequence similarity was seen within the kinetoplastid *Trypanosoma brucei* 65 %, *Mycobacterium tuberculosis* which is also an intracellular parasite like *Leishmania*, however, did not show any presence of GGTase-II enzyme on data mining. Available literature indicated that *Mycobacterium* is predicted to express secreted effectors containing CAAX motifs that may be prenylated by host protein prenyltransferases [44]. While *Drosophila melanogaster*, *Homo sapiens* and *Plasmodium falciparum* were showed 52, 48 and 29 % sequence similarity to GGTase-II (β-subunit) of *Leishmania donovani* respectively (Fig. 8).

Template for GGTase-II (β-subunit) of *Leishmania donovani* was selected on the basis of sequence similarity and lowest E-value. Blastp analysis against PDB database, GGTase-II (β-subunit) of *Rattus norvegicus* shows 48 % identity and minimum E-value with GGTase-II (β-subunit) of *Leishmania donovani* and this template was further used for molecular modeling studies.

**Fig. 9:**
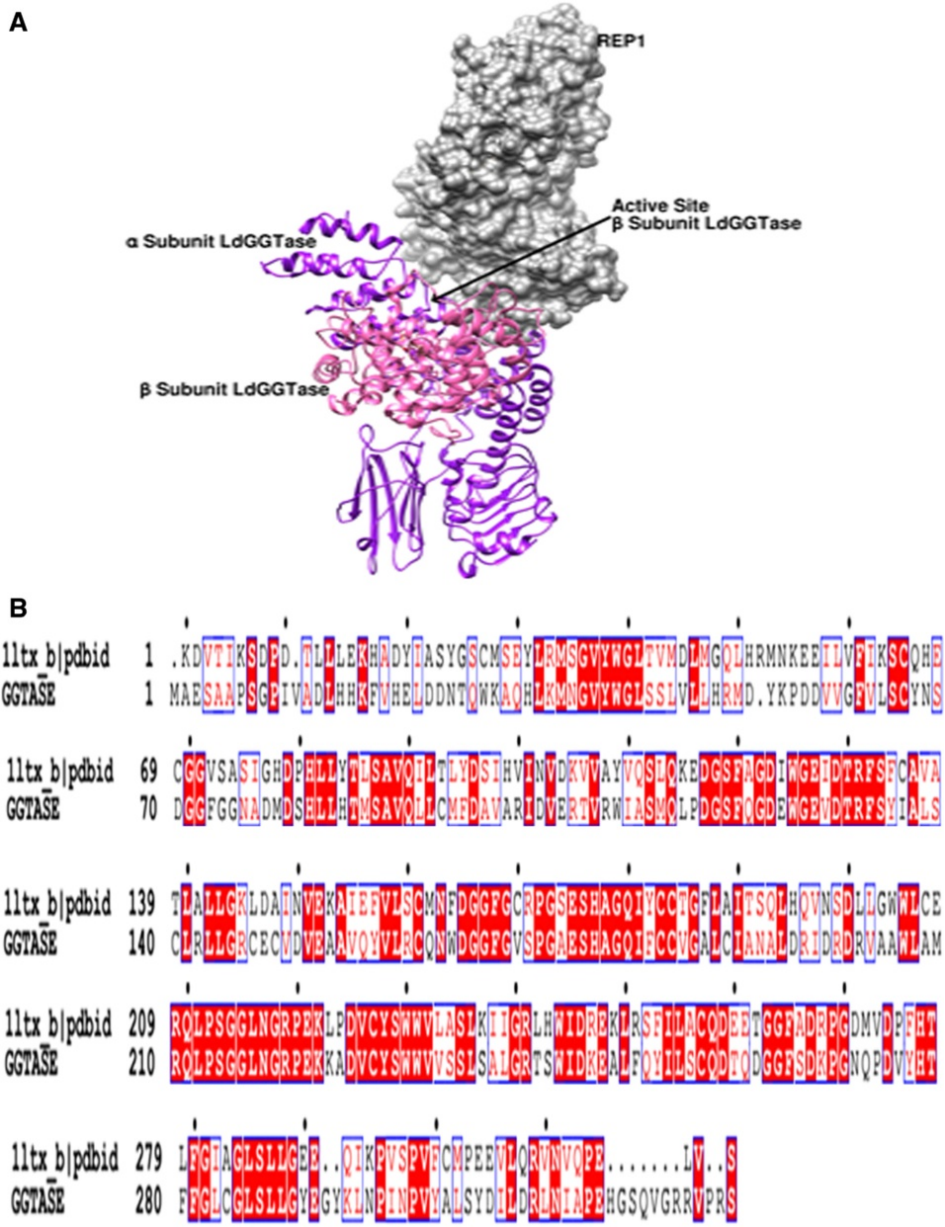
**a** Showed interaction between Rab Escort protein-1 (REP- 1) and geranylgeranyl transferase-II enzyme of *Leishmania donovani* (LdGGTase-II). REP- 1 is displayed in surface representation and colored in grey. α and β-subunits of LdGGTase-II are displayed in ribbon representation and colored in *purple* and *magenta* respectively, whereas arrow indicated active site of β-subunit of LdGGTase-II. **b** Sequence alignment of the template (PDB ID 11tx_b) and the protein (β-subunit of LdGGTase-II). Residues highlighted in *red* correspond to identical/conserved residues, while residues in *red text* are similar in these two proteins

**Fig. 10:**
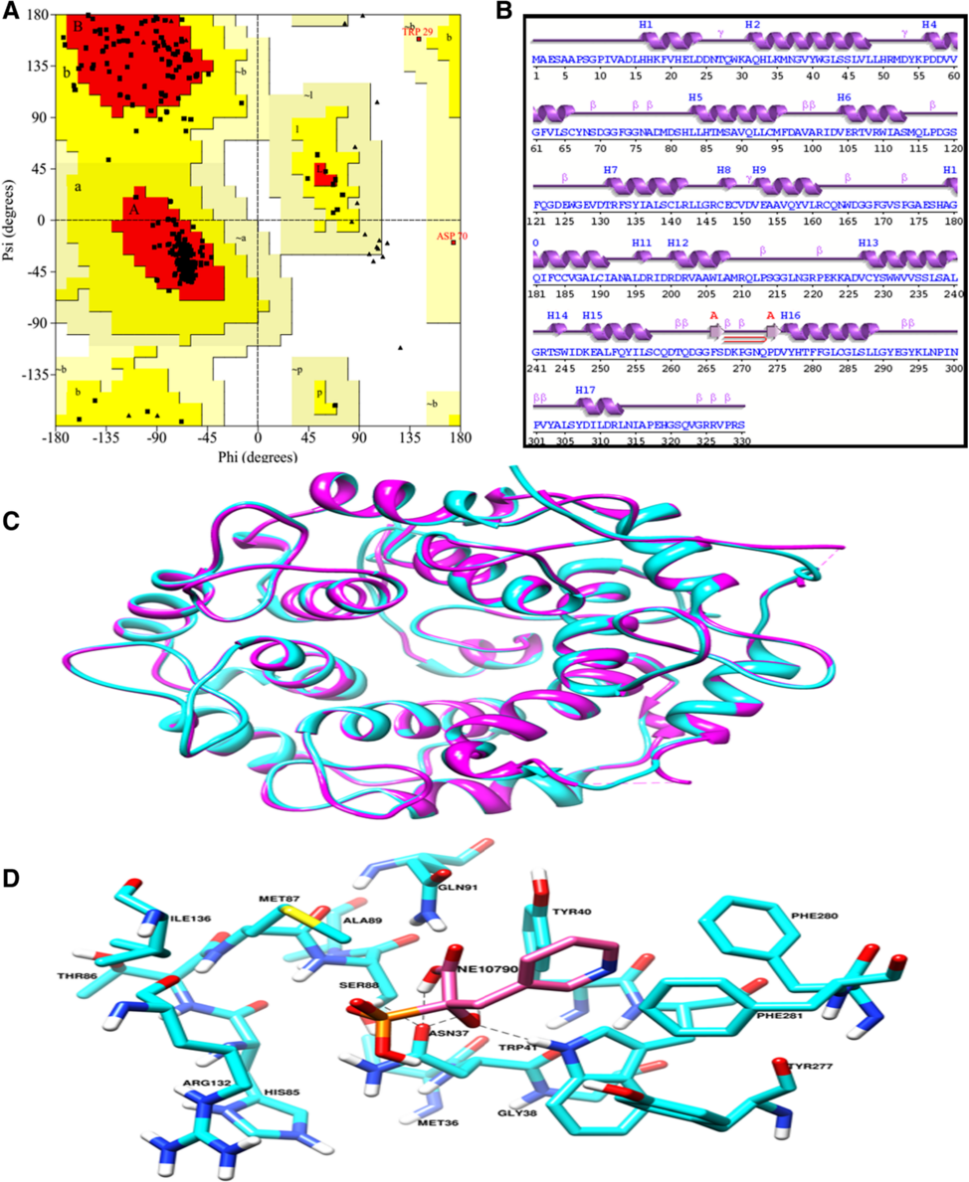
**a** Ramachandran plot of the homology-modeled structure of *Leishmania donovani* Rabgeranylgeranyltransferase. The different colored areas indicate “disallowed” (*white*), “generously allowed” (*light yellow*), “additional allowed” (*yellow*), and “most favored” (*red*) regions. **b** The wiring diagram of β-subunit of LdGGTase-II was performed using the PDBsum server (http://www.ebi.ac.uk/thornton-srv/databases/pdbsum/). **c** Superimposed structures of *L. donovani* RabGGTase (*cyan*) with template 1LTX (*magenta*). **d** The binding mode of inhibitor NE10790 (Pink) in the docked complex of *L. donovani* RabGGTase (Cyan)

We have ascertained that the β-subunit of GGTase-II in both *Leishmania* and *Leptomonas* lie in close proximity done through Neighbor-Joining method (Fig. 5). Therefore the β-subunit emerges as the excellent target for compounds inhibiting parasite activity in clinical cases of co-infections. The crystal structure of Rab escort protein-1 in complex with geranylgeranyltransferase-II (Fig. 9a) and isoprenoid from *Rattus* showed 48 % of sequence similarity (Fig. 9b) with β-subunit of *Leishmania* GGTase-II thus selected as the template for homology modeling [45]. Ten models were generated with Modeller. The validation of the resulting models was performed using the SAVS server and the best validated model was selected [46]. In the selected model, the majority of the residues (89.4 %) occupy the most favored region of Ramachandran Plot generated by PROCHECK and 9.9, 0.7 and 0.0 % residues lie in additional allowed region (yellow), generously allowed (light yellow) and disallowed (white) region respectively (Fig. 10a). The wiring diagram of β-subunit of LdGGTase-II was performed using the PDBsum server (http://www.ebi.ac.uk/thornton-srv/databases/pdbsum/); a schematic diagram showed the protein’s secondary structure elements (alpha-helices and beta-sheets) together with various structural motifs such as beta- and gamma-turns, and beta-hairpins. Helices are labeled as H1, H2, etc. (Fig. 10b). The superimposition of the modeled protein (*L. donovani* RabGGTase) with template 1LTX shows an RMSD of 0.205 Å (Fig. 10c). The farnesyl binding site was selected as the active site for docking of the inhibitor. The NE10790 has a docking score of −6.10 kcal/mol. The inhibitor is involved in formation of three hydrogen bonds with Asn37 and one hydrogen bond with Trp41 as shown by binding mode (Fig. 10d).

## Conclusions

**Fig. 11:**
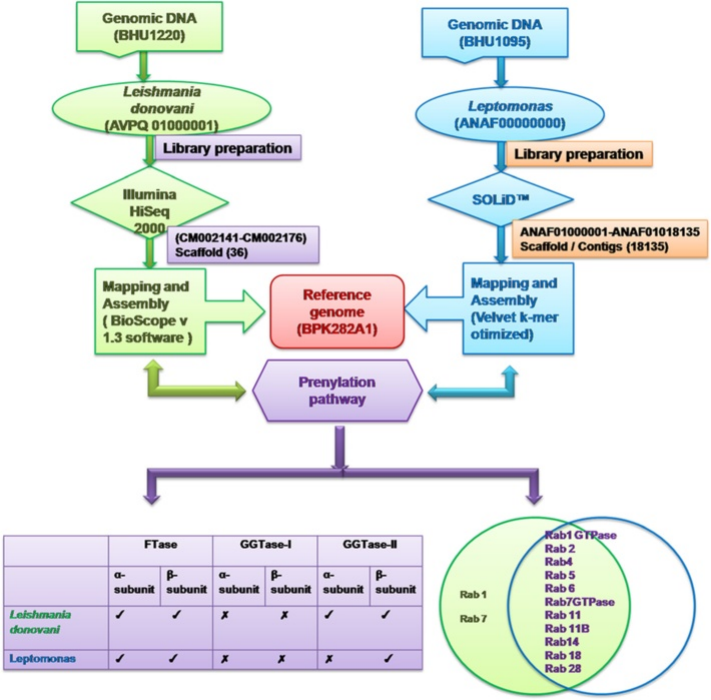
Data mining of the prenylation pathway from the genomes of *L. donovani* and *Leptomonas*

By data mining of the prenylation pathway from the genomes of *L. donovani* and *Leptomonas*, we have tried to analyse the evolutionary and functional similarities between both these kinetoplastids (Fig. 11). When compared to Rab proteins defined and elucidated in *Homo sapiens*, the presence of Rabs in *Leishmania* suggests that *Leishmania* had a sophisticated endo membrane system, which provides its ability to survive in various intracellular and extracellular stages. This detailed analysis of Rab proteins has provided information of degree of complexity of exo and endocytic trafficking in this organism. The presence of SDR motif known to be associated with drug resistance in several Rab genes of *Leishmania* indicates that as the parasite evolved from monogenetic to digenetic lifestyle it acquired capabilities to evade drug action for its survival in host. The disruption of prenylation pathway of the parasite using highly specific inhibitors will disable the parasite to reside and survive intracellularly within host phagosome microenvironment and prevent it from modulating host defense pathways for its survival.

## Methods

### Whole genome sequence

*L. major* and *L. infantum* genomes are available at (www.ncbi.nlm.nih.gov) with accession number GCA_000002725.2 and GCA_000002875.2 respectively.

Whole genome sequence (WGS) of kinetoplastid species has been deposited by us for *Leishmania donovani* (BHU 1220, AVPQ01000001) http://www.ncbi.nlm.nih.gov/Traces/wgs/?val=AVPQ01000001and *Leptomonas* (BHU 1095, ANAF00000000.1) (http://www.ncbi.nlm.nih.gov/Traces/wgs/?val=ANAF01#contigs) [1].

### Kinetoplastid genome phylogeny

Following genomes sequences were retrieved from TritrypDB database.

#### *Crithidia fasciculata*

(http://tritrypdb.org/tritrypdb/showApplication.do).

#### *Leishmania aethiopica*

(http://tritrypdb.org/tritrypdb/showApplication.do).

#### *Leishmania amazonensis*

(http://tritrypdb.org/tritrypdb/showApplication.do).

#### *Leishmania arabica*

(http://tritrypdb.org/tritrypdb/showApplication.do).

#### *Leishmania braziliensis*

(http://tritrypdb.org/tritrypdb/showApplication.do).

#### *Leishmania enriettii*

(http://tritrypdb.org/tritrypdb/showApplication.do).

#### *Leishmania gerbilli*

(http://tritrypdb.org/tritrypdb/showApplication.do).

#### *Leishmania donovani*

(http://tritrypdb.org/tritrypdb/showApplication.do).

#### *Leishmania major*

(http://tritrypdb.org/tritrypdb/showApplication.do).

#### *Leishmania mexicana*

(http://tritrypdb.org/tritrypdb/showApplication.do).

#### *L. sp. MAR LEM2494*

(http://tritrypdb.org/tritrypdb/showApplication.do).

#### *Leishmania tarentolae*

(http://tritrypdb.org/tritrypdb/showApplication.do).

#### *Leishmania tropica*

(http://tritrypdb.org/tritrypdb/showApplication.do).

#### *Leishmania turanica*

(http://tritrypdb.org/tritrypdb/showApplication.do).

#### *Leptomonas seymouri*

(http://tritrypdb.org/tritrypdb/showApplication.do).

For whole-genome multiple sequence alignments of all these 15 genomes were carried out using Mugsy (http://mugsy.sf.net). Core segments of the alignment that are shared among all isolates included in the analysis were identified and concatenated. The phylogenetic analysis was carried out by considering BHU1220 as reference to the other 14 genome using Phylomark and dendrogram.

### Database extraction of geranylgeranyltransferase from whole genome sequence

Rab geranylgeranyltransferase is a heterodimer, consisting of α and β subunits. When we searched for GGTase-I and FTase –I or its homologous sequences in the genomes of *L. major, L. infantum, L. donovani* and *Leptomonas,* we found no GGTase-I sequences (EC. 2.5.1.59) in any of the *Leishmania* species but putative farnesyltransferase sequences (EC. 2.5.1.58) of *Leishmania major* was found on chromosome 31 (Lmj F31.2940) with accession no. XP_001685284, and in *Leishmania infantum* also it is located on chromosome 31 (LinJ_31_3050) with accession no. XP_00146718.

When we searched for GGTase-II and its homologous sequences in *Leishmania*, we found GGTasae-II equences but it was putative and only β-subunit was annotated. In *Leishmania major* it was located on chromosome 34 (Lmj F34.4030) with accession no. XP_001686510, in *Leishmania infantum* it was located on chromosome 34 (LinJ_34_3860) with accession no. XP_001468743 and in *Leishmania donovani* it was also located on chromosome 34 (LdBPK_343860) with accession no. XP_003864545. In *Leishmania donovani* there is only one protein of 330 amino acids (Accession no. XP_003864545), annotated as putative Rab-GGTase.

For annotation of α-subunit of GGTase-II *of L. donovani*, it was located on chromosome 33 with accession no XM_001468149.

When we searched for GGTase-II and its homologous sequences in *Leptomonas* genome we found no α-subunit of GGTase-II but β-subunit of GGTase-II was obtained. To annotate the β-subunit of GGTase-II in *Leptomonas*, the DNA sequence was retrieved from the contig (genes_5507) of its genome and was put in the translate tools of Expasy. Open reading frame of the β-subunit of GGTase-II from *Leptomonas* was thus obtained. Blastp analysis of β-subunit of GGTase-II ORF was then done by NCBI and we obtained the sequences producing significant alignment with *L. panamensis* (XP_010698581), *L. braziliensis* (XP_001564646) and *L. major* (XP_001686510).

Multiple sequence alignments of farnesyltransferase and geranylgeranyltransferase were performed with ClustalW. The evolutionary history was inferred using the Neighbor-Joining method [47]. Phylogenetic reconstruction was done using 1000 bootstraps and evolutionary distances were computed using the JTT matrix-based method [48, 49], are in the units of the number of amino acid substitutions per site. The analysis involved 8 amino acid sequences. All positions containing gaps and missing data were eliminated. Evolutionary analysis were conducted in MEGA5 [50]. Trees were drawn using in FigTree v. 1.0.

### Rab sequence mining in *L. major* and *L. infantum*

Initially in first set we extracted all the annotated *H. sapiens* Rab sequences reported previously from NCBI [17]. Based upon their annotation they were grouped in families, subfamilies and isoforms. Then each Rab protein sequence was used as query to blastp against the NCBI protein database of *L. major* and *L. infantum*, a cutoff for inclusion of protein in dataset was set to be having > 30 % similarity with the query sequence. For generation of second dataset all the sequences retrieved in first dataset being already well annotated as a Rab protein of a family were grouped and Clustal was done to find the conserved domains in these Rab families [51].

Further analysis was done by NCBI conserved domain search (http://www.ncbi.nlm.nih.gov/Structure/cdd/wrpsb.cgi) and motif scan (http://hits.isb-sib.ch/cgi-bin/PFSCAN) server to check the conserved domains in these proteins. Based on these results a consensus profile sequence for each family of the Rab was generated which included conserved domains, these consensus sequences were used as query sequence in PSI-BLAST against *L. major* and *L. infantum* database at NCBI to retrieve Rab family sequences. Similarly a cutoff of >30 % similarity was set for including sequence in the dataset. Sequences in all the datasets were having similarity more than cut off value were retrieved in fasta format, they were checked for redundancy and duplication or incorrect annotation with rejection of sequence from dataset if it showed 100 % redundancy with any other sequence. The protein datasets were treated separately using the jalview software [52]. Then all the Rab groups in the datasets were compared with each other for redundancy at sequence level. The sequences thus obtained were then fed to the PrePS server (http://mendel.imp.ac.at/PrePS/index.html) [53] to check that proteins can be predicted to be of Rab family and gets prenylated by GGTase-II enzyme.

Sequence similarity between Rab proteins of *L. major* and *L. infantum* was calculated by MatGAT (Matrix Global Alignment Tool. 2.02) software. It is a simple, easy to use computer application that generates similarity/identity matrices for protein sequences without needing pre-alignment of the data [54].

### Rab sequence mining in *L. donovani* and *Leptomonas*

The Rabs in *L. donovani* were retrieved from its genome based on homology search for the corresponding protein sequences from the reference genome of *L. infantum.* 17 Rabs were identified from its genome as Rabs and Rabs like sequences; whereas 13 out of 17 Rabs were fully annotated (Additional file 2: Table S2).

The Rabs in *Leptomonas* were retrieved from the contigs of its genome and were based on homology search for the corresponding protein sequences from the reference genome of *L. infantum*. To annotate the Rabs in these contigs, the DNA sequence of these contigs was put in the translate tools of Expasy. Open reading frames of the Rab proteins of *Leptomonas* were thus obtained. Blastp analysis of these Rab ORFs was then done by NCBI and we obtained the Rab sequences producing significant alignment with *L. infantum*. We were able to retrieve fully annotated 11 Rabs out of 53 contigs (Additional file 2: Table S3).

### Multiple sequence alignment and phylogenetic reconstruction

The protein sequences were aligned by using ClustalW algorithm in MEGALIGN, DNASTAR package (DNASTAR, Inc. Madison WI, USA). Phylogenetic trees were generated with MEGALIGN which implements a Maximum Likelihood probabilistic model, using standard parameters (gap penalty of 15 and gap length penalty of 6.66) and 1000 bootstraps. The graphically enhanced alignment was obtained using ESPript 2.2 server. The branch lengths are drawn proportional to the evolutionary distances. The numbers at the nodes show the bootstrap support for that node.

### Molecular modeling studies of GGTase-II with specific drug target molecule

Since Rab proteins play critical roles in all aspects of intracellular membrane trafficking, this makes them particularly attractive therapeutic targets in diseases. Proper function of Rab proteins depends upon correct membrane localization which is achieved through geranylgeranylation. The quest to determine agents which disrupt Rab geranylgeranylation therefore represents an intriguing therapeutic strategy by which to induce cellular stress and apoptosis. Two lines of attack may achieve diminished cellular levels of Rab geranylgeranylation. One approach would be to deplete cells of the isoprenoid substrate geranylgeranyl diphosphate. This might be achieved by inhibition of any of the individual steps in the isoprenoid biosynthetic pathway that lead to geranylgeranyldiphosphate (GGDP), from inhibition of HMGCoA reductase by a statin, to inhibition of GGPP synthase. This suggests that a long, lipophilic tail can enhance the potency of potential GGTase-II inhibitors, presumably through interaction with the enzyme site that holds the tail of the natural substrate geranylgeranyl diphosphate. The second direct strategy involves inhibition of GGTase-II itself. While inhibitors of GGTase-II are rare, a few have been reported [55]. In silico analysis we used NE10790 for its ability to inhibit *Leishmania* GGTase-II using GGPP, Rab6 substrate and REP. Our results show that NE10790 is an active compound which bears a geranylgeranyl chain.

Our search for *Leishmania* GGTase-II crystal structure in databases showed that there are no crystal structures of *Leishmania* GGTase-II for structural studies, so we carried out the exercise of molecular modeling of *Leishmania donovani* GGTase-II. Three-dimensional (3D) homology model of *Leishmania donovani* geranylgeranyltransferase-II (LdGGTase-II) was generated by comparative modeling using MODELLER 9v8 [56] with crystal structure of Rab escort protein-1 in complex with Rab geranylgeranyltransferase and isoprenoid from *Rattus norvegicus* (PDB ID-1LTX) was selected as a template for structure modeling. ClustalW server was used to align the query sequence with the template sequence [57]. The graphically enhanced alignment was generated with the help of ESPript 2.2 server [58]. All models were assessed stereo-chemically by PROCHECK [46]. All the graphical visualization and image production were performed using Chimera [59]. The docking studies were performed using Autodock4.2 software [60]. The structure of the inhibitor NE10790 was drawn using sketch module of Sybyl7.1 and geometry was optimized by performing energy minimization using MMFF94 force field with 1000 iterations (Sybyl, version 7.1; Tripos, Inc.: St. Louis, MO, 2005).

### Availability of supporting data

Whole genome sequence (WGS) of *Leishmania donovani* (BHU 1220, AVPQ01000001) and *Leptomonas* (BHU 1095, ANAF00000000.1) supporting the results of this article is available at http://www.ncbi.nlm.nih.gov/Traces/wgs/?val=AVPQ01000001http://www.ncbi.nlm.nih.gov/Traces/wgs/?val=ANAF01#contigs.

All the supporting data are included as additional files.

## Additional files

Additional file 1: Figure S1. Multiple sequence alignment of FTase (α-subunit) of *Leishmania donovani* (accession number XP_003862625.1, putative protein) with FTase (α-subunit) of *Leishmania major* (accession number XP_003722277.1, putative protein), *Leishmania infantum* (accession number XP_001466722.1, putative protein) and its sister *Leptomonas* (contig_2654, putative protein) showing conserved regions (red colored) based on percentage identity. **Figure S2.** Multiple sequence alignment of FTase (β-subunit) of *Leishmania donovani* (accession number XP_003861732.1) with FTase (β-subunit) of *Leishmania major* (accession number XP_001684151), *Leishmania infantum* (accession number XP_001470492) and its sister *Leptomonas* (contig_1135) showing conserved regions (red colored) based on percentage identity. **Figure S3.** Multiple sequence alignment of GGTase-II (α-subunit) of *Leishmania donovani* (accession number XM_001468149) with GGTase-II (α-subunit) of *Leishmania major* (accession number XM_001685808) and *Leishmania infantum* (accession number XP_001468186) showing conserved regions (red colored) based on percentage identity. Its sister *Leptomonas* has no α -subunit of GGTase-II. **Figure S4.** Multiple sequence alignment of GGTase-II (β-subunit) of *Leishmania donovani* (accession number XP_003864545) with GGTase-II (β-subunit) of *Leishmania major* (accession number XP_001686510) and *Leishmania infantum* (accession number XP_001468743) showing conserved regions (red colored) based on percentage identity. **Figure S5.** Alignment of various *Leishmania donovani* Rab protein sequences showing sequence similarities and domain conservation among them colored red based in their percentage similarity. **Figure S6.** Alignment of various *Leptomonas* Rab protein sequences showing sequence similarities and domain conservation among them colored red based in their percentage similarity. **Figure S7.** Phylogenetic relationship among Rab sequences of *L. major, L. infantum, L. donovani* and *Leptomonas* which is based on multiple sequence alignment (ClustalW algorithm in MEGALIGN with 1000 bootstraps). **Figure S8.** Circos plot was generated using Circos version 0.60, which is used to determine the similarity between Rab genes of *Leishmania donovani* and its sister *Leptomonas*. Where cyan color for all Rabs protein obtained from *Leptomonas* genome and green color for all Rabs protein obtained from *Leishmania donovani* genome. Other colors for each different Rabs. (ZIP 81526 kb) Additional file 2: Table S1.
*L. major* and *L. infantum* Rabs with their accession numbers, gene IDs and chromosome location. **Table S2.** Rab GTPase sequences identified from *Leishmania donovani*. **Table S3.** Rab GTPase sequences identified from *Leptomonas.* **Table S4.** (**A**) Heat-map depicting percent identity between *L. donovani* and (**B**) *Leptomonas*. (DOC 102 kb)

## Abbreviations

WGS: Whole genome sequencing
GGTase: Geranylgeranyltransferase
FTase: Farnesyltransferase
REP: Rab escort protein
PDB: Protein Data Bank
LECA: Last eukaryotic common ancestor
AIP: Actin interacting protein
SDR: Short-chain dehydrogenase/ reductase
MDR: Multi-drug resistance
ORF: Open reading frame
