# Supplementary figures and images for: Evolutionary comparison of prenylation pathway in kinetoplastid Leishmania and its sister Leptomonas
Source: BMC Evol Biol. 2015 Nov 21;15:261. doi: 10.1186/s12862-015-0538-3 (PMC4654808; doi:10.1186/s12862-015-0538-3)

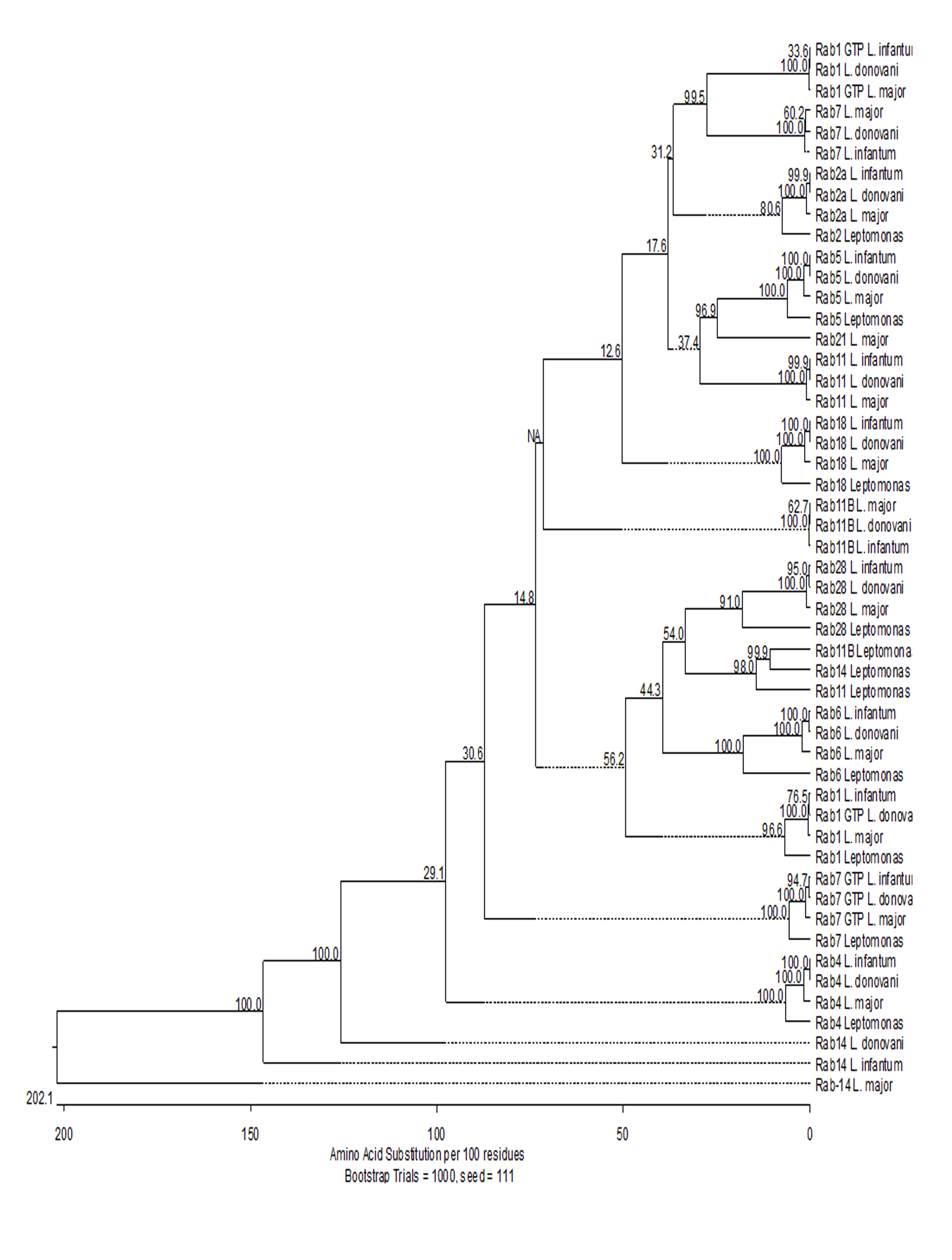

Supplement: Additional file 1: Figure S1. — Multiple sequence alignment of FTase (α-subunit) of Leishmania donovani (accession number XP_003862625.1, putative protein) with FTase (α-subunit) of Leishmania major (accession number XP_003722277.1, putative protein), Leishmania infantum (accession number XP_001466722.1, putative protein) and its sister Leptomonas (contig_2654, putative protein) showing conserved regions (red colored) based on percentage identity. Figure S2. Multiple sequence alignment of FTase (β-subunit) of Leishmania donovani (accession number XP_003861732.1) with FTase (β-subunit) of Leishmania major (accession number XP_001684151), Leishmania infantum (accession number XP_001470492) and its sister Leptomonas (contig_1135) showing conserved regions (red colored) based on percentage identity. Figure S3. Multiple sequence alignment of GGTase-II (α-subunit) of Leishmania donovani (accession number XM_001468149) with GGTase-II (α-subunit) of Leishmania major (accession number XM_001685808) and Leishmania infantum (accession number XP_001468186) showing conserved regions (red colored) based on percentage identity. Its sister Leptomonas has no α -subunit of GGTase-II. Figure S4. Multiple sequence alignment of GGTase-II (β-subunit) of Leishmania donovani (accession number XP_003864545) with GGTase-II (β-subunit) of Leishmania major (accession number XP_001686510) and Leishmania infantum (accession number XP_001468743) showing conserved regions (red colored) based on percentage identity. Figure S5. Alignment of various Leishmania donovani Rab protein sequences showing sequence similarities and domain conservation among them colored red based in their percentage similarity. Figure S6. Alignment of various Leptomonas Rab protein sequences showing sequence similarities and domain conservation among them colored red based in their percentage similarity. Figure S7. Phylogenetic relationship among Rab sequences of L. major, L. infantum, L. donovani and Leptomonas which is based on multiple sequence alignmen [file 12862_2015_538_MOESM1_ESM.zip › Additional file 1 Figure S7.tif]

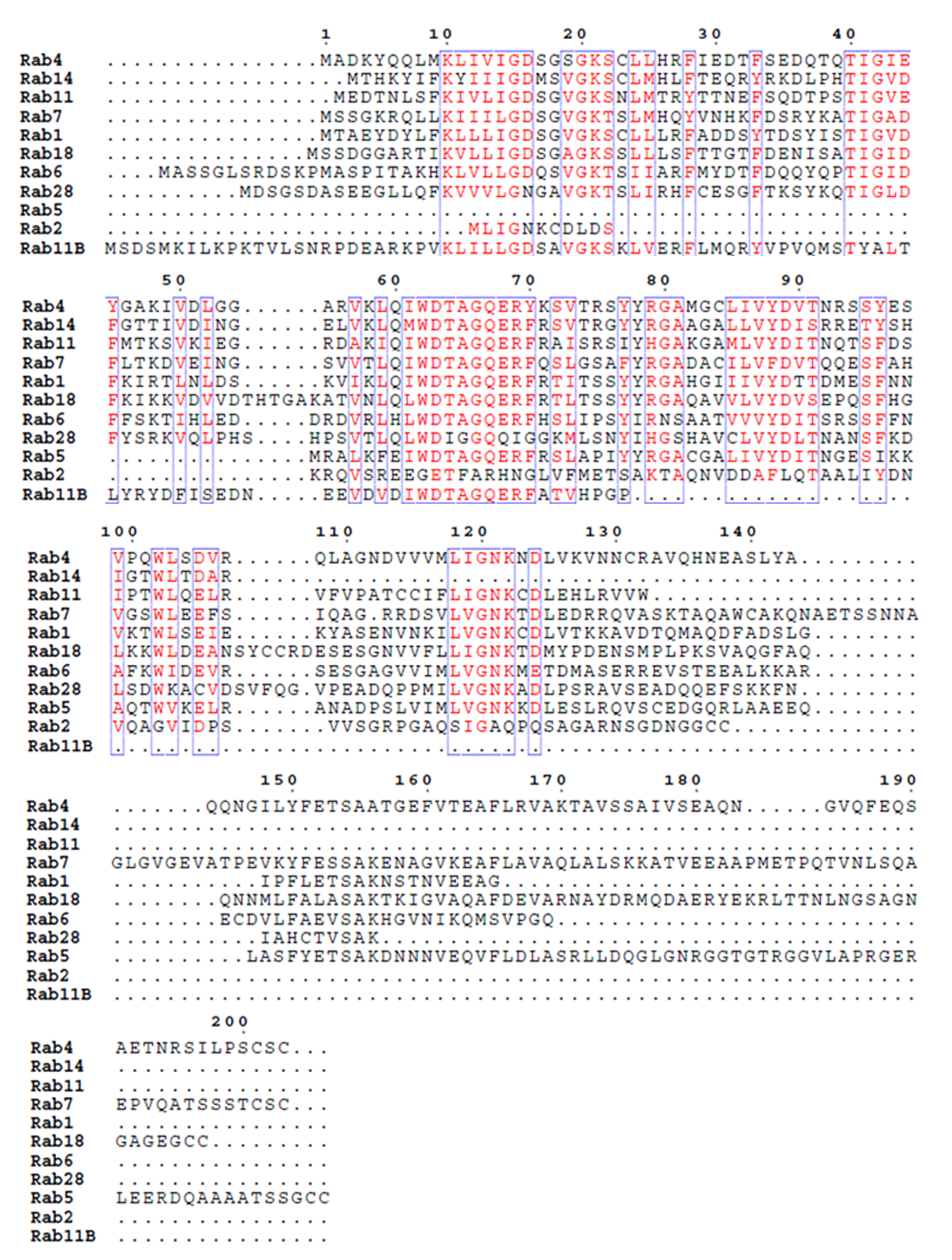

Supplement: Additional file 1: Figure S1. — Multiple sequence alignment of FTase (α-subunit) of Leishmania donovani (accession number XP_003862625.1, putative protein) with FTase (α-subunit) of Leishmania major (accession number XP_003722277.1, putative protein), Leishmania infantum (accession number XP_001466722.1, putative protein) and its sister Leptomonas (contig_2654, putative protein) showing conserved regions (red colored) based on percentage identity. Figure S2. Multiple sequence alignment of FTase (β-subunit) of Leishmania donovani (accession number XP_003861732.1) with FTase (β-subunit) of Leishmania major (accession number XP_001684151), Leishmania infantum (accession number XP_001470492) and its sister Leptomonas (contig_1135) showing conserved regions (red colored) based on percentage identity. Figure S3. Multiple sequence alignment of GGTase-II (α-subunit) of Leishmania donovani (accession number XM_001468149) with GGTase-II (α-subunit) of Leishmania major (accession number XM_001685808) and Leishmania infantum (accession number XP_001468186) showing conserved regions (red colored) based on percentage identity. Its sister Leptomonas has no α -subunit of GGTase-II. Figure S4. Multiple sequence alignment of GGTase-II (β-subunit) of Leishmania donovani (accession number XP_003864545) with GGTase-II (β-subunit) of Leishmania major (accession number XP_001686510) and Leishmania infantum (accession number XP_001468743) showing conserved regions (red colored) based on percentage identity. Figure S5. Alignment of various Leishmania donovani Rab protein sequences showing sequence similarities and domain conservation among them colored red based in their percentage similarity. Figure S6. Alignment of various Leptomonas Rab protein sequences showing sequence similarities and domain conservation among them colored red based in their percentage similarity. Figure S7. Phylogenetic relationship among Rab sequences of L. major, L. infantum, L. donovani and Leptomonas which is based on multiple sequence alignmen [file 12862_2015_538_MOESM1_ESM.zip › Additional file 1 Figure S6.tif]

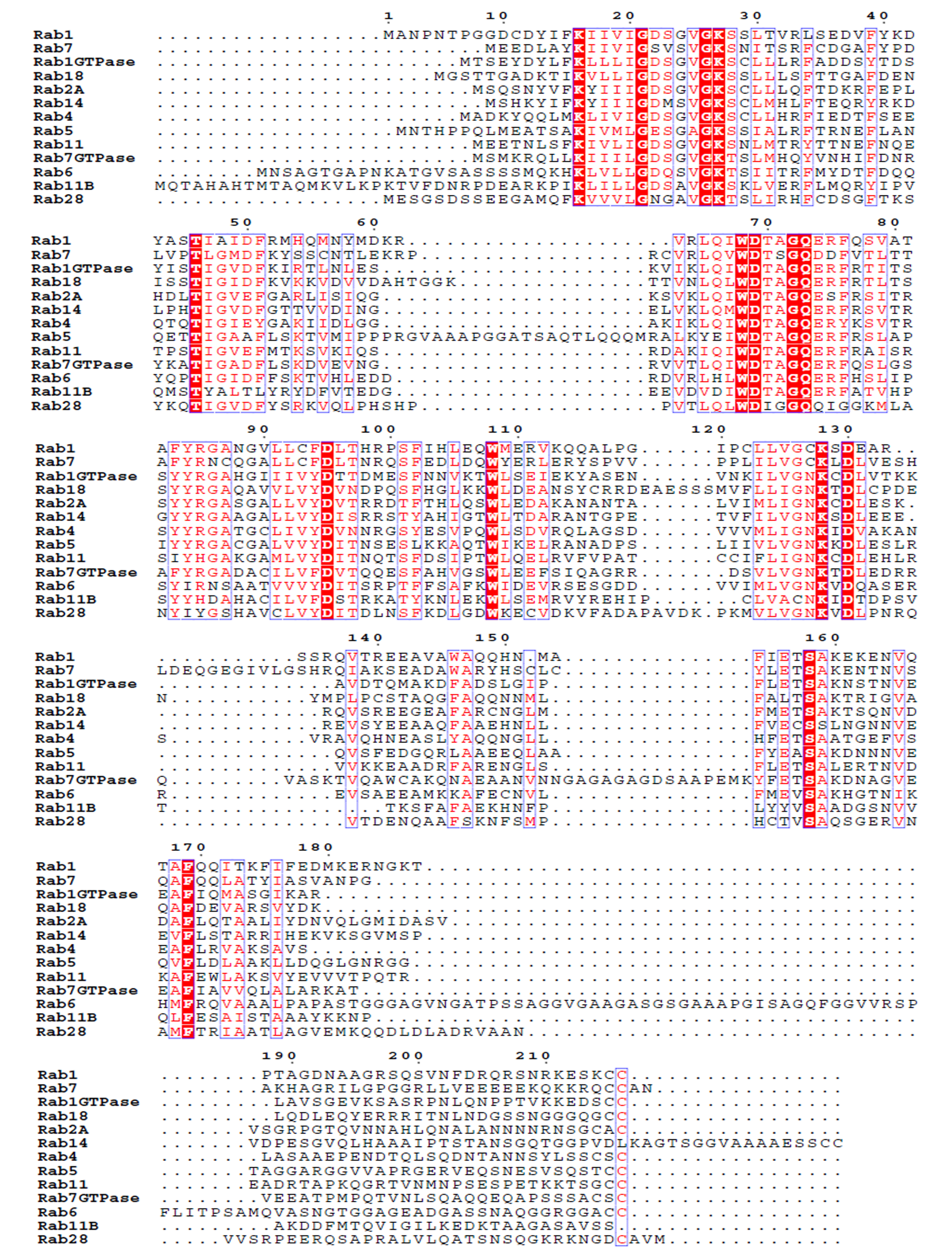

Supplement: Additional file 1: Figure S1. — Multiple sequence alignment of FTase (α-subunit) of Leishmania donovani (accession number XP_003862625.1, putative protein) with FTase (α-subunit) of Leishmania major (accession number XP_003722277.1, putative protein), Leishmania infantum (accession number XP_001466722.1, putative protein) and its sister Leptomonas (contig_2654, putative protein) showing conserved regions (red colored) based on percentage identity. Figure S2. Multiple sequence alignment of FTase (β-subunit) of Leishmania donovani (accession number XP_003861732.1) with FTase (β-subunit) of Leishmania major (accession number XP_001684151), Leishmania infantum (accession number XP_001470492) and its sister Leptomonas (contig_1135) showing conserved regions (red colored) based on percentage identity. Figure S3. Multiple sequence alignment of GGTase-II (α-subunit) of Leishmania donovani (accession number XM_001468149) with GGTase-II (α-subunit) of Leishmania major (accession number XM_001685808) and Leishmania infantum (accession number XP_001468186) showing conserved regions (red colored) based on percentage identity. Its sister Leptomonas has no α -subunit of GGTase-II. Figure S4. Multiple sequence alignment of GGTase-II (β-subunit) of Leishmania donovani (accession number XP_003864545) with GGTase-II (β-subunit) of Leishmania major (accession number XP_001686510) and Leishmania infantum (accession number XP_001468743) showing conserved regions (red colored) based on percentage identity. Figure S5. Alignment of various Leishmania donovani Rab protein sequences showing sequence similarities and domain conservation among them colored red based in their percentage similarity. Figure S6. Alignment of various Leptomonas Rab protein sequences showing sequence similarities and domain conservation among them colored red based in their percentage similarity. Figure S7. Phylogenetic relationship among Rab sequences of L. major, L. infantum, L. donovani and Leptomonas which is based on multiple sequence alignmen [file 12862_2015_538_MOESM1_ESM.zip › Additional file 1 Figure S5.tif]

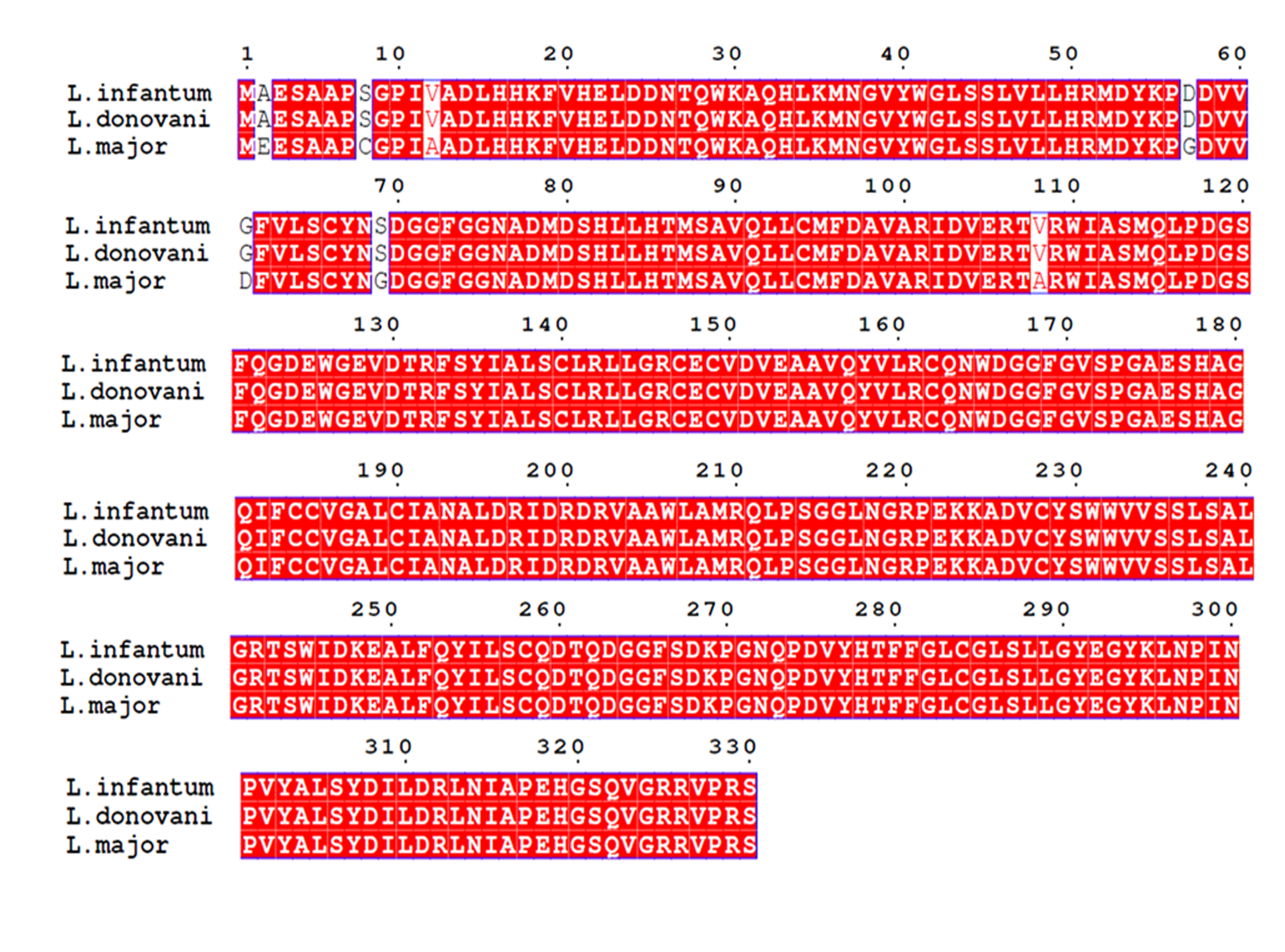

Supplement: Additional file 1: Figure S1. — Multiple sequence alignment of FTase (α-subunit) of Leishmania donovani (accession number XP_003862625.1, putative protein) with FTase (α-subunit) of Leishmania major (accession number XP_003722277.1, putative protein), Leishmania infantum (accession number XP_001466722.1, putative protein) and its sister Leptomonas (contig_2654, putative protein) showing conserved regions (red colored) based on percentage identity. Figure S2. Multiple sequence alignment of FTase (β-subunit) of Leishmania donovani (accession number XP_003861732.1) with FTase (β-subunit) of Leishmania major (accession number XP_001684151), Leishmania infantum (accession number XP_001470492) and its sister Leptomonas (contig_1135) showing conserved regions (red colored) based on percentage identity. Figure S3. Multiple sequence alignment of GGTase-II (α-subunit) of Leishmania donovani (accession number XM_001468149) with GGTase-II (α-subunit) of Leishmania major (accession number XM_001685808) and Leishmania infantum (accession number XP_001468186) showing conserved regions (red colored) based on percentage identity. Its sister Leptomonas has no α -subunit of GGTase-II. Figure S4. Multiple sequence alignment of GGTase-II (β-subunit) of Leishmania donovani (accession number XP_003864545) with GGTase-II (β-subunit) of Leishmania major (accession number XP_001686510) and Leishmania infantum (accession number XP_001468743) showing conserved regions (red colored) based on percentage identity. Figure S5. Alignment of various Leishmania donovani Rab protein sequences showing sequence similarities and domain conservation among them colored red based in their percentage similarity. Figure S6. Alignment of various Leptomonas Rab protein sequences showing sequence similarities and domain conservation among them colored red based in their percentage similarity. Figure S7. Phylogenetic relationship among Rab sequences of L. major, L. infantum, L. donovani and Leptomonas which is based on multiple sequence alignmen [file 12862_2015_538_MOESM1_ESM.zip › Additional file 1 Figure S4.tif]

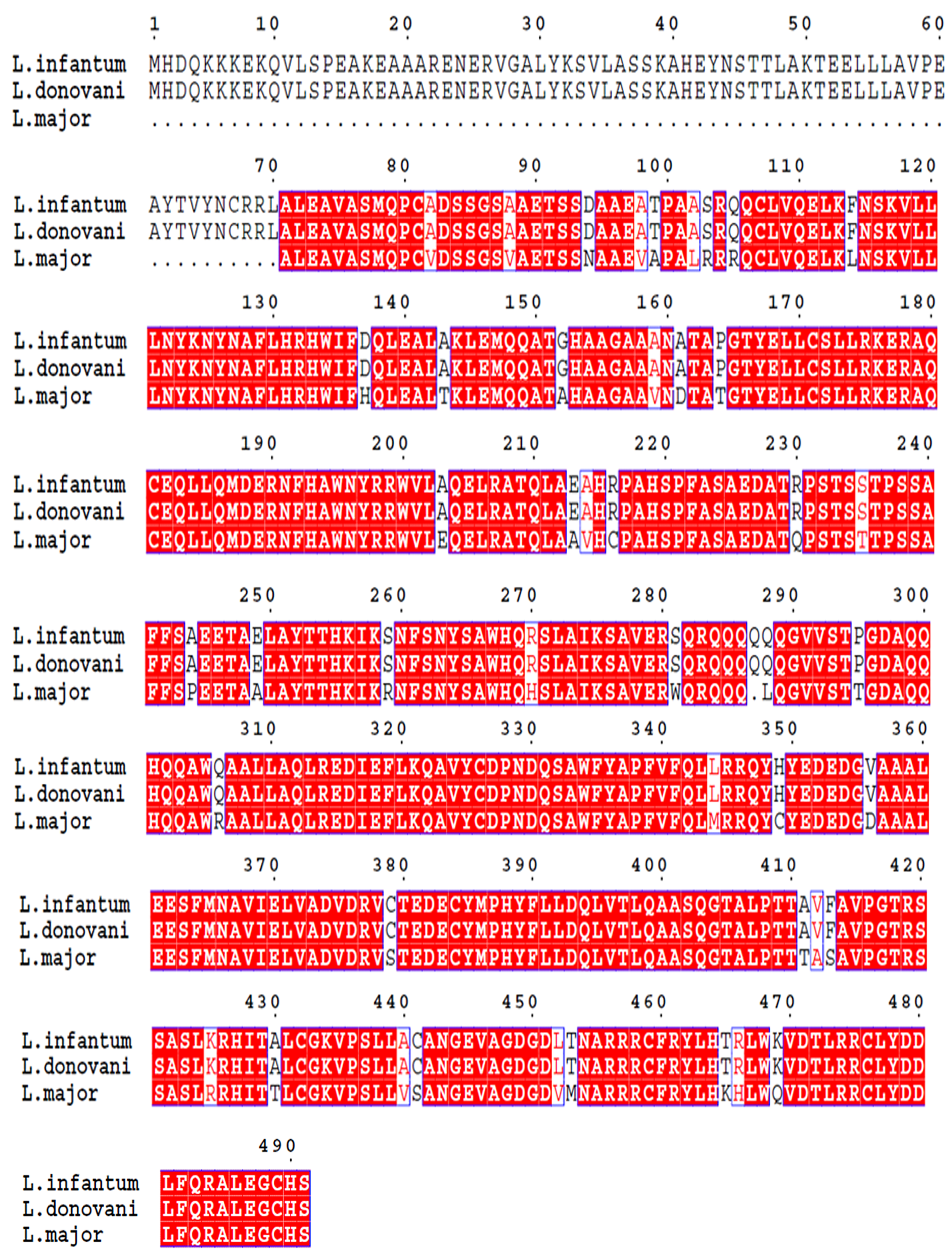

Supplement: Additional file 1: Figure S1. — Multiple sequence alignment of FTase (α-subunit) of Leishmania donovani (accession number XP_003862625.1, putative protein) with FTase (α-subunit) of Leishmania major (accession number XP_003722277.1, putative protein), Leishmania infantum (accession number XP_001466722.1, putative protein) and its sister Leptomonas (contig_2654, putative protein) showing conserved regions (red colored) based on percentage identity. Figure S2. Multiple sequence alignment of FTase (β-subunit) of Leishmania donovani (accession number XP_003861732.1) with FTase (β-subunit) of Leishmania major (accession number XP_001684151), Leishmania infantum (accession number XP_001470492) and its sister Leptomonas (contig_1135) showing conserved regions (red colored) based on percentage identity. Figure S3. Multiple sequence alignment of GGTase-II (α-subunit) of Leishmania donovani (accession number XM_001468149) with GGTase-II (α-subunit) of Leishmania major (accession number XM_001685808) and Leishmania infantum (accession number XP_001468186) showing conserved regions (red colored) based on percentage identity. Its sister Leptomonas has no α -subunit of GGTase-II. Figure S4. Multiple sequence alignment of GGTase-II (β-subunit) of Leishmania donovani (accession number XP_003864545) with GGTase-II (β-subunit) of Leishmania major (accession number XP_001686510) and Leishmania infantum (accession number XP_001468743) showing conserved regions (red colored) based on percentage identity. Figure S5. Alignment of various Leishmania donovani Rab protein sequences showing sequence similarities and domain conservation among them colored red based in their percentage similarity. Figure S6. Alignment of various Leptomonas Rab protein sequences showing sequence similarities and domain conservation among them colored red based in their percentage similarity. Figure S7. Phylogenetic relationship among Rab sequences of L. major, L. infantum, L. donovani and Leptomonas which is based on multiple sequence alignmen [file 12862_2015_538_MOESM1_ESM.zip › Additional file 1 Figure S3.tif]

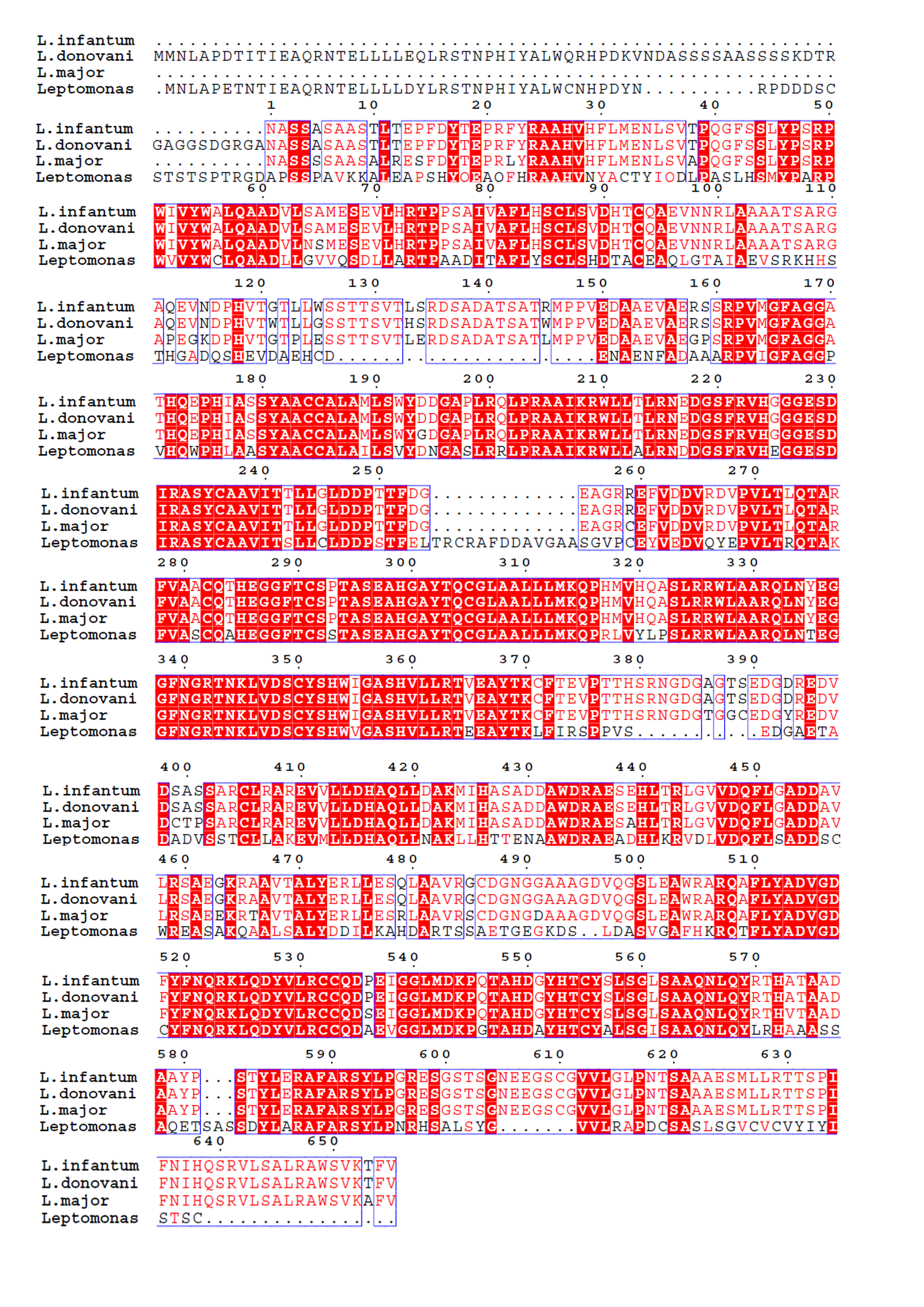

Supplement: Additional file 1: Figure S1. — Multiple sequence alignment of FTase (α-subunit) of Leishmania donovani (accession number XP_003862625.1, putative protein) with FTase (α-subunit) of Leishmania major (accession number XP_003722277.1, putative protein), Leishmania infantum (accession number XP_001466722.1, putative protein) and its sister Leptomonas (contig_2654, putative protein) showing conserved regions (red colored) based on percentage identity. Figure S2. Multiple sequence alignment of FTase (β-subunit) of Leishmania donovani (accession number XP_003861732.1) with FTase (β-subunit) of Leishmania major (accession number XP_001684151), Leishmania infantum (accession number XP_001470492) and its sister Leptomonas (contig_1135) showing conserved regions (red colored) based on percentage identity. Figure S3. Multiple sequence alignment of GGTase-II (α-subunit) of Leishmania donovani (accession number XM_001468149) with GGTase-II (α-subunit) of Leishmania major (accession number XM_001685808) and Leishmania infantum (accession number XP_001468186) showing conserved regions (red colored) based on percentage identity. Its sister Leptomonas has no α -subunit of GGTase-II. Figure S4. Multiple sequence alignment of GGTase-II (β-subunit) of Leishmania donovani (accession number XP_003864545) with GGTase-II (β-subunit) of Leishmania major (accession number XP_001686510) and Leishmania infantum (accession number XP_001468743) showing conserved regions (red colored) based on percentage identity. Figure S5. Alignment of various Leishmania donovani Rab protein sequences showing sequence similarities and domain conservation among them colored red based in their percentage similarity. Figure S6. Alignment of various Leptomonas Rab protein sequences showing sequence similarities and domain conservation among them colored red based in their percentage similarity. Figure S7. Phylogenetic relationship among Rab sequences of L. major, L. infantum, L. donovani and Leptomonas which is based on multiple sequence alignmen [file 12862_2015_538_MOESM1_ESM.zip › Additional file 1 Figure S2.tif]

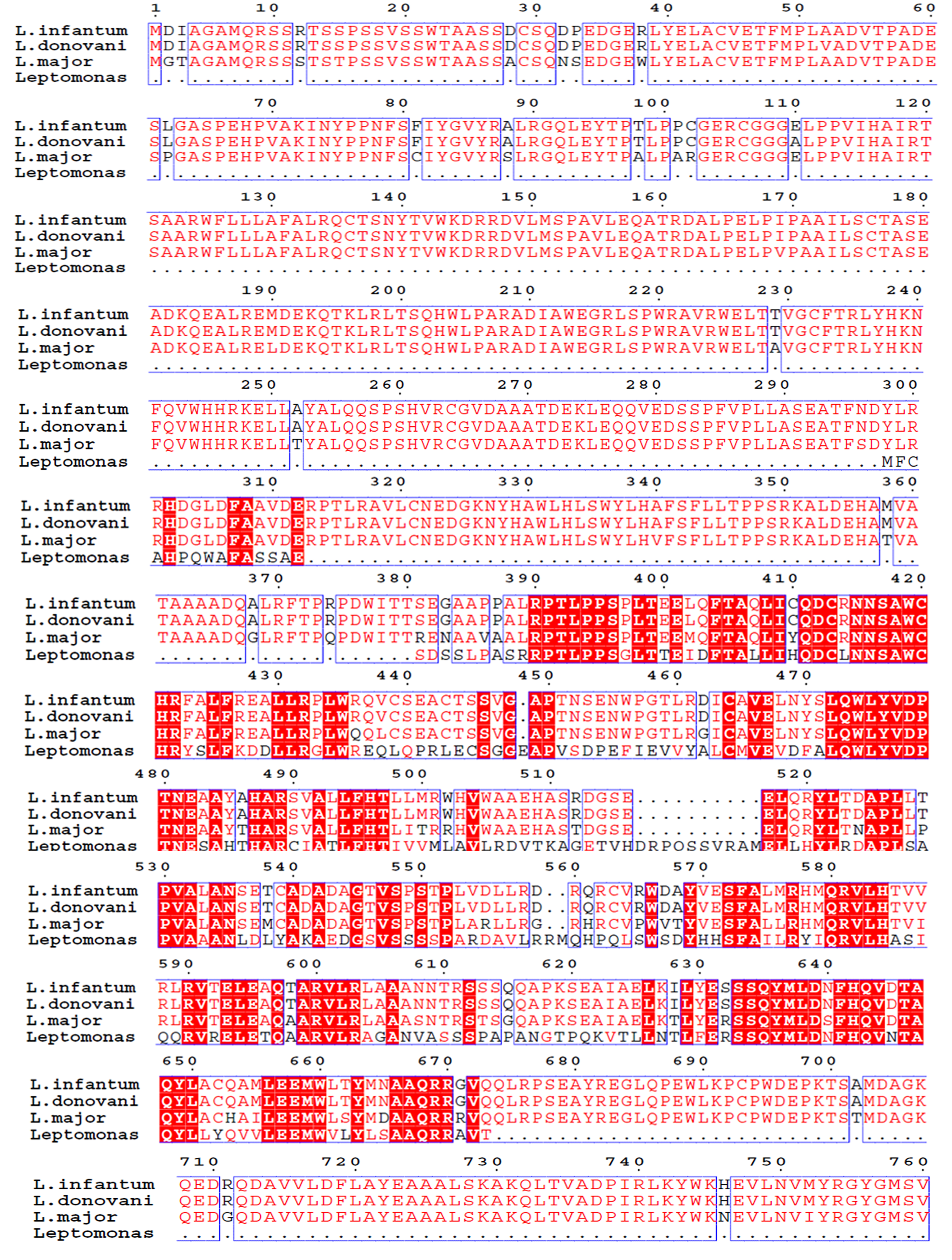

Supplement: Additional file 1: Figure S1. — Multiple sequence alignment of FTase (α-subunit) of Leishmania donovani (accession number XP_003862625.1, putative protein) with FTase (α-subunit) of Leishmania major (accession number XP_003722277.1, putative protein), Leishmania infantum (accession number XP_001466722.1, putative protein) and its sister Leptomonas (contig_2654, putative protein) showing conserved regions (red colored) based on percentage identity. Figure S2. Multiple sequence alignment of FTase (β-subunit) of Leishmania donovani (accession number XP_003861732.1) with FTase (β-subunit) of Leishmania major (accession number XP_001684151), Leishmania infantum (accession number XP_001470492) and its sister Leptomonas (contig_1135) showing conserved regions (red colored) based on percentage identity. Figure S3. Multiple sequence alignment of GGTase-II (α-subunit) of Leishmania donovani (accession number XM_001468149) with GGTase-II (α-subunit) of Leishmania major (accession number XM_001685808) and Leishmania infantum (accession number XP_001468186) showing conserved regions (red colored) based on percentage identity. Its sister Leptomonas has no α -subunit of GGTase-II. Figure S4. Multiple sequence alignment of GGTase-II (β-subunit) of Leishmania donovani (accession number XP_003864545) with GGTase-II (β-subunit) of Leishmania major (accession number XP_001686510) and Leishmania infantum (accession number XP_001468743) showing conserved regions (red colored) based on percentage identity. Figure S5. Alignment of various Leishmania donovani Rab protein sequences showing sequence similarities and domain conservation among them colored red based in their percentage similarity. Figure S6. Alignment of various Leptomonas Rab protein sequences showing sequence similarities and domain conservation among them colored red based in their percentage similarity. Figure S7. Phylogenetic relationship among Rab sequences of L. major, L. infantum, L. donovani and Leptomonas which is based on multiple sequence alignmen [file 12862_2015_538_MOESM1_ESM.zip › Additional file 1 Figure S1.tif]

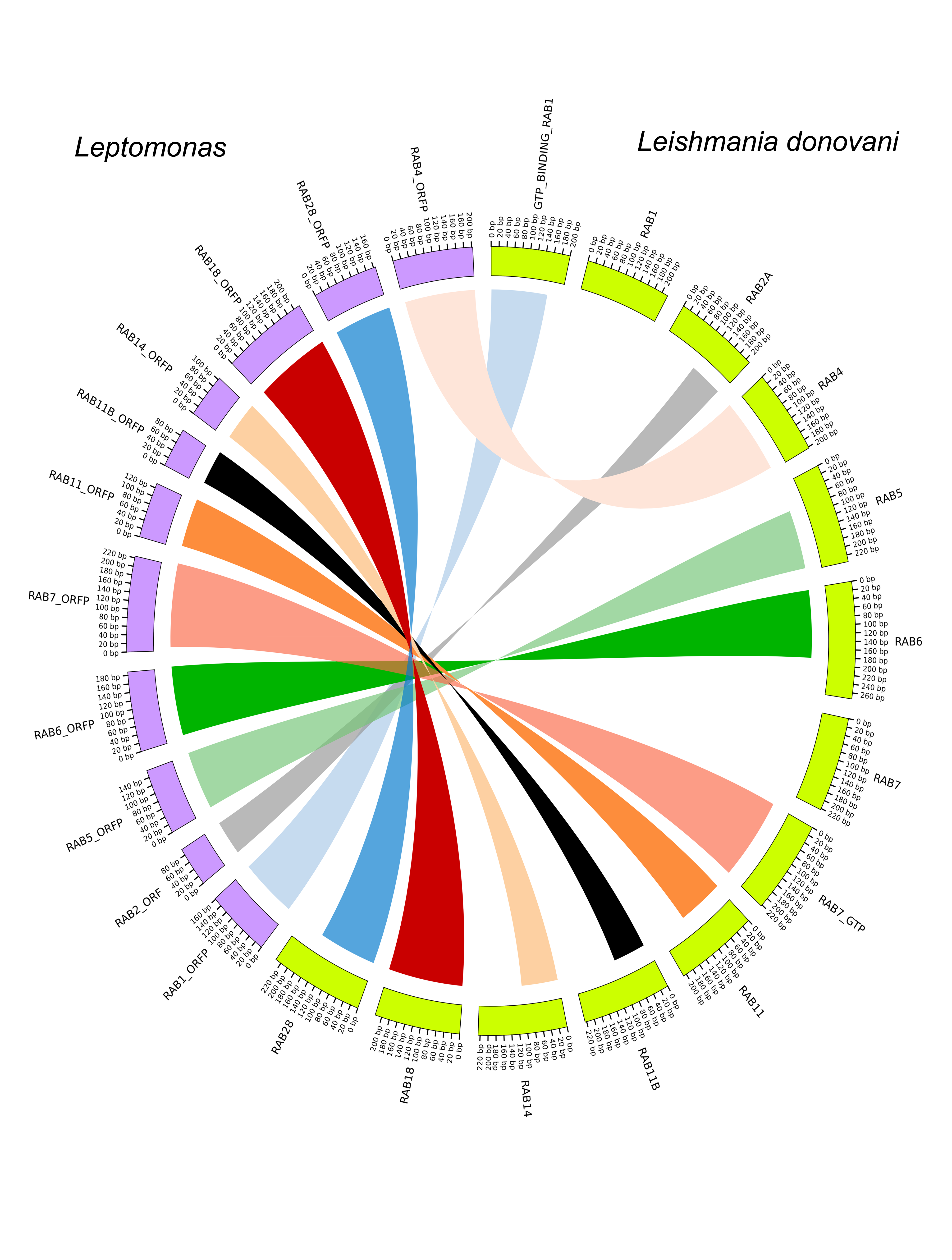

Supplement: Additional file 1: Figure S1. — Multiple sequence alignment of FTase (α-subunit) of Leishmania donovani (accession number XP_003862625.1, putative protein) with FTase (α-subunit) of Leishmania major (accession number XP_003722277.1, putative protein), Leishmania infantum (accession number XP_001466722.1, putative protein) and its sister Leptomonas (contig_2654, putative protein) showing conserved regions (red colored) based on percentage identity. Figure S2. Multiple sequence alignment of FTase (β-subunit) of Leishmania donovani (accession number XP_003861732.1) with FTase (β-subunit) of Leishmania major (accession number XP_001684151), Leishmania infantum (accession number XP_001470492) and its sister Leptomonas (contig_1135) showing conserved regions (red colored) based on percentage identity. Figure S3. Multiple sequence alignment of GGTase-II (α-subunit) of Leishmania donovani (accession number XM_001468149) with GGTase-II (α-subunit) of Leishmania major (accession number XM_001685808) and Leishmania infantum (accession number XP_001468186) showing conserved regions (red colored) based on percentage identity. Its sister Leptomonas has no α -subunit of GGTase-II. Figure S4. Multiple sequence alignment of GGTase-II (β-subunit) of Leishmania donovani (accession number XP_003864545) with GGTase-II (β-subunit) of Leishmania major (accession number XP_001686510) and Leishmania infantum (accession number XP_001468743) showing conserved regions (red colored) based on percentage identity. Figure S5. Alignment of various Leishmania donovani Rab protein sequences showing sequence similarities and domain conservation among them colored red based in their percentage similarity. Figure S6. Alignment of various Leptomonas Rab protein sequences showing sequence similarities and domain conservation among them colored red based in their percentage similarity. Figure S7. Phylogenetic relationship among Rab sequences of L. major, L. infantum, L. donovani and Leptomonas which is based on multiple sequence alignmen [file 12862_2015_538_MOESM1_ESM.zip › Additional file 1 Figure S8.tif]
