# Supplementary material for: Evolutionary comparison of prenylation pathway in kinetoplastid Leishmania and its sister Leptomonas
Source: BMC Evol Biol. 2015 Nov 21;15:261. doi: 10.1186/s12862-015-0538-3 (PMC4654808; doi:10.1186/s12862-015-0538-3)
Supplement: Additional file 2: Table S1. — L. major and L. infantum Rabs with their accession numbers, gene IDs and chromosome location. Table S2. Rab GTPase sequences identified from Leishmania donovani. Table S3. Rab GTPase sequences identified from Leptomonas. Table S4. (A) Heat-map depicting percent identity between L. donovani and (B) Leptomonas. (DOC 102 kb) [file 12862_2015_538_MOESM2_ESM.doc]

**Additional file 2:**

**Table S1** *L. major* and *L. infantum* Rabs with their accession numbers, gene IDs and chromosome location

|  | ***L. major* Rabs** | | | | ***L. infantum* Rabs** | | | |
| --- | --- | --- | --- | --- | --- | --- | --- | --- |
| S. No. | Annotated Name | Acc. No. | Gene ID | Chromosome  Location | Annotated Name | Acc. No. | Gene ID | Chromosome  Location |
| 1 | Putative Rab-1 | XP_003721882.1 | [12982665](http://www.ncbi.nlm.nih.gov/sites/entrez?db=gene&cmd=Retrieve&dopt=full_report&list_uids=12982665) | Chr-27 | Putative Rab-1 | XP_003392649.1 | [10966266](http://www.ncbi.nlm.nih.gov/sites/entrez?db=gene&cmd=Retrieve&dopt=full_report&list_uids=10966266) | Chr-27 |
| 2 | Putative Rab-1 GTP | XP_001681450.1 | [5649721](http://www.ncbi.nlm.nih.gov/sites/entrez?db=gene&cmd=Retrieve&dopt=full_report&list_uids=5649721) | Chr-10 | Putative Rab-1 GTP | XP_001463769.1 | [5067037](http://www.ncbi.nlm.nih.gov/sites/entrez?db=gene&cmd=Retrieve&dopt=full_report&list_uids=5067037) | Chr-10 |
| 3 | Putative Rab-2a | XP_001685525.1 | [5656322](http://www.ncbi.nlm.nih.gov/sites/entrez?db=gene&cmd=Retrieve&dopt=full_report&list_uids=5656322) | Chr-32 | Putative Rab-2a | XP_001467894.1 | [5071957](http://www.ncbi.nlm.nih.gov/sites/entrez?db=gene&cmd=Retrieve&dopt=full_report&list_uids=5071957) | Chr-32 |
| 4 | Putative Rab-4 | XP_001685364.1 | [5656160](http://www.ncbi.nlm.nih.gov/sites/entrez?db=gene&cmd=Retrieve&dopt=full_report&list_uids=5656160) | Chr-32 | Putative Rab-4 | XP_001467752.1 | [5071810](http://www.ncbi.nlm.nih.gov/sites/entrez?db=gene&cmd=Retrieve&dopt=full_report&list_uids=5071810) | Chr-32 |
| 5 | Putative Rab-5 | XP_001682519.1 | 5651041 | Chr-18 | Putative Rab-5 | XP_001464928.1 | [5068336](http://www.ncbi.nlm.nih.gov/sites/entrez?db=gene&cmd=Retrieve&dopt=full_report&list_uids=5068336) | Chr-18 |
| 6 | Rab-6-like | XP_003721632.1 | [12981564](http://www.ncbi.nlm.nih.gov/sites/entrez?db=gene&cmd=Retrieve&dopt=full_report&list_uids=12981564) | Chr-02 | Putative Rab-6 | XP_001462715.1 | [5066187](http://www.ncbi.nlm.nih.gov/sites/entrez?db=gene&cmd=Retrieve&dopt=full_report&list_uids=5066187) | Chr-02 |
| 7 | Putative Rab-7 GTP | XP_001682495.1 | [5651017](http://www.ncbi.nlm.nih.gov/sites/entrez?db=gene&cmd=Retrieve&dopt=full_report&list_uids=5651017) | Chr-18 | Putative Rab-7 GTP | XP_003392405.1 | [10966032](http://www.ncbi.nlm.nih.gov/sites/entrez?db=gene&cmd=Retrieve&dopt=full_report&list_uids=10966032) | Chr-18 |
| 8 | Putative Rab-11 | XP_001681425.1 | [5649694](http://www.ncbi.nlm.nih.gov/sites/entrez?db=gene&cmd=Retrieve&dopt=full_report&list_uids=5649694) | Chr-10 | Putative Rab-11 | XP_001463744.1 | [5067124](http://www.ncbi.nlm.nih.gov/sites/entrez?db=gene&cmd=Retrieve&dopt=full_report&list_uids=5067124) | Chr-10 |
| 9 | Putative Rab-11B | XP_001685503.1 | [5656300](http://www.ncbi.nlm.nih.gov/sites/entrez?db=gene&cmd=Retrieve&dopt=full_report&list_uids=5656300) | Chr-32 | Putative Rab-11B | XP_001467868.1 | [5071929](http://www.ncbi.nlm.nih.gov/sites/entrez?db=gene&cmd=Retrieve&dopt=full_report&list_uids=5071929) | Chr-32 |
| 10 | Putative Rab-14 | XP_001680962.1 | [5649214](http://www.ncbi.nlm.nih.gov/sites/entrez?db=gene&cmd=Retrieve&dopt=full_report&list_uids=5649214) | Chr-07 | Putative Rab-14 | XP_001463263.1 | [5066595](http://www.ncbi.nlm.nih.gov/sites/entrez?db=gene&cmd=Retrieve&dopt=full_report&list_uids=5066595) | Chr-07 |
| 11 | Putative Rab-18 | XP_001685944.1 | 5654607 | Chr-33 | Putative Rab-18 | XP_001468265.1 | [5072339](http://www.ncbi.nlm.nih.gov/sites/entrez?db=gene&cmd=Retrieve&dopt=full_report&list_uids=5072339) | Chr-33 |
| 12 | Putative Rab-21 | XP_001683014.1 | [5651617](http://www.ncbi.nlm.nih.gov/sites/entrez?db=gene&cmd=Retrieve&dopt=full_report&list_uids=5651617) | Chr-21 | Putative Rab-21 | ---- | --- | --- |
| 13 | Putative Rab-28 | XP_001684775.1 | [5653714](http://www.ncbi.nlm.nih.gov/sites/entrez?db=gene&cmd=Retrieve&dopt=full_report&list_uids=5653714) | Chr-30 | Putative Rab-28 | XP_001467017.1 | [5071061](http://www.ncbi.nlm.nih.gov/sites/entrez?db=gene&cmd=Retrieve&dopt=full_report&list_uids=5071061) | Chr-30 |
| 14 | Putative Rab-7 | XP_001681451.1 | [5649722](http://www.ncbi.nlm.nih.gov/sites/entrez?db=gene&cmd=Retrieve&dopt=full_report&list_uids=5649722) | Chr-10 | Putative Rab-7 | XP_001463770.1 | [5067010](http://www.ncbi.nlm.nih.gov/sites/entrez?db=gene&cmd=Retrieve&dopt=full_report&list_uids=5067010) | Chr-10 |

**Table S2** Rab GTPase sequences identified from *Leishmania donovani*

| **Gene Ids** | **Description** |
| --- | --- |
| LdBPK_101260 | small GTP-binding protein Rab7, putative |
| LdBPK_100960 | small GTP-binding protein Rab11, putative |
| LdBPK_270620 | small GTP-binding protein Rab1, putative |
| LdBPK_301710 | small GTP-binding protein Rab28, putative |
| LdBPK_331940 | small GTP-binding protein Rab18, putative |
| LdBPK_020230 | small GTP binding protein Rab6-like protein |
| LdBPK_070610 | Ras-related protein Rab-14, putative |
| LdBPK_101250 | Rab1 small GTP-binding protein, putative |
| LdBPK_181140 | Ras-related protein Rab-5, putative |
| LdBPK_180890 | Rab7 GTP binding protein, putative |
| LdBPK_321930 | Rab11B GTPase, putative |
| LdBPK_320520 | Ras-related Rab-4, putative |
| LdBPK_322160 | Ras-related protein Rab-2a, putative |

**Table S3** Rab GTPase sequences identified from *Leptomonas*

| **Gene Ids** | **Description** |
| --- | --- |
| gene_643 | Ras-related protein Rab-2a |
| gene_1715 | small GTP-binding protein Rab28 |
| gene_1795 | small GTP-binding protein Rab18 |
| gene_2455 | Ras-related protein Rab-5 |
| gene_2598 | small GTP-binding protein Rab7 |
| gene_2977 | Rab11b GTPase |
| gene_3933 | Rab11b GTPase |
| gene_6205 | Rab7 GTP binding partial |
| gene_6637 | Rab11b GTPase |
| gene_6663 | small GTP -binding protein Rab7 |
| gene_7212 | small GTP-binding protein Rab18 |
| gene_8530 | small GTP-binding protein Rab1 |
| gene_8743 | Ras-related protein Rab-5 |
| gene_8904 | Rab1 small GTP-binding protein |
| gene_9238 | Ras-related protein Rab-5 |
| gene_9603 | small GTP-binding protein Rab11 |
| gene_10825 | Rab7 GTPbinding protein |
| gene_13186 | Ras-related protein Rab-2a |
| gene_13583 | Ras-related protein Rab-2a |
| gene_15416 | Small GTP binding protein Rab6-like protein |
| gene_16984 | Ras-related Rab-4 |

**Table S4** Heat-map depicting percent identity between **[A]** *L. donovani* and **[B]** *Leptomonas*

**[A]** *L. donovani*

*
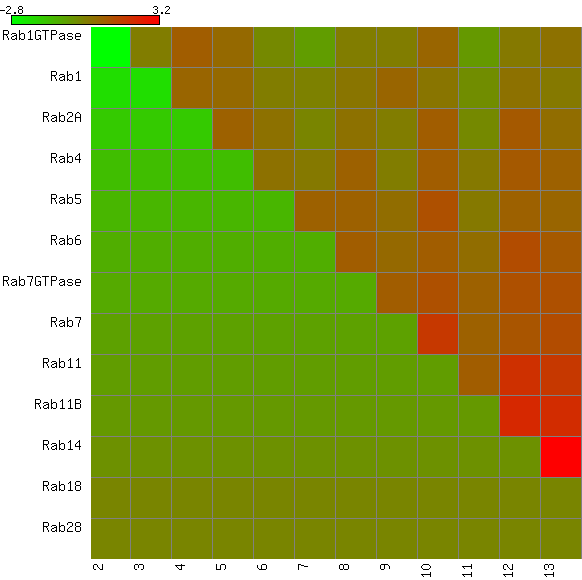
*

**[B]** *Leptomonas.*

*
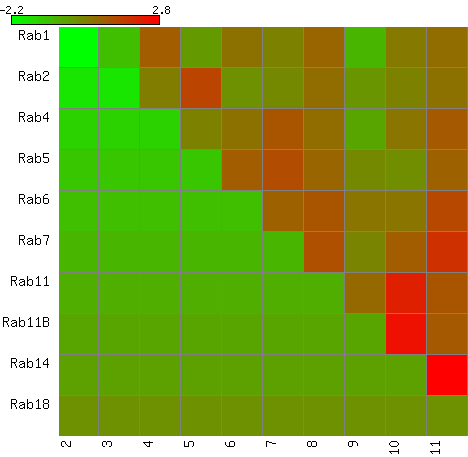
*
